# Elongating maize root: zone-specific combinations of polysaccharides from type I and type II primary cell walls

**DOI:** 10.1038/s41598-020-67782-0

**Published:** 2020-07-02

**Authors:** Liudmila V. Kozlova, Alsu R. Nazipova, Oleg V. Gorshkov, Anna A. Petrova, Tatyana A. Gorshkova

**Affiliations:** Laboratory of Plant Cell Growth Mechanisms, Kazan Institute of Biochemistry and Biophysics, FRC Kazan Scientific Center of RAS, Kazan, Russian Federation

**Keywords:** Plant development, Plant physiology

## Abstract

The dynamics of cell wall polysaccharides may modulate the cell wall mechanics and thus control the expansion growth of plant cells. The unique composition of type II primary cell wall characteristic of grasses suggests that they employ specific mechanisms for cell enlargement. We characterized the transcriptomes in five zones along maize root, clustered the expression of genes for numerous glycosyltransferases and performed extensive immunohistochemical analysis to relate the changes in cell wall polysaccharides to critical stages of cell development in Poaceae. Specific patterns of cell wall formation differentiate the initiation, realization and cessation of elongation growth. Cell walls of meristem and early elongation zone represent a mixture of type I and type II specific polysaccharides. Xyloglucans and homogalacturonans are synthesized there actively together with mixed-linkage glucans and glucuronoarabinoxylans. Rhamnogalacturonans-I with the side-chains of branched 1,4-galactan and arabinan persisted in cell walls throughout the development. Thus, the machinery to generate the type I primary cell wall constituents is completely established and operates. The expression of glycosyltransferases responsible for mixed-linkage glucan and glucuronoarabinoxylan synthesis peaks at active or late elongation. These findings widen the number of jigsaw pieces which should be put together to solve the puzzle of grass cell growth.

## Introduction

**Figure 1:**
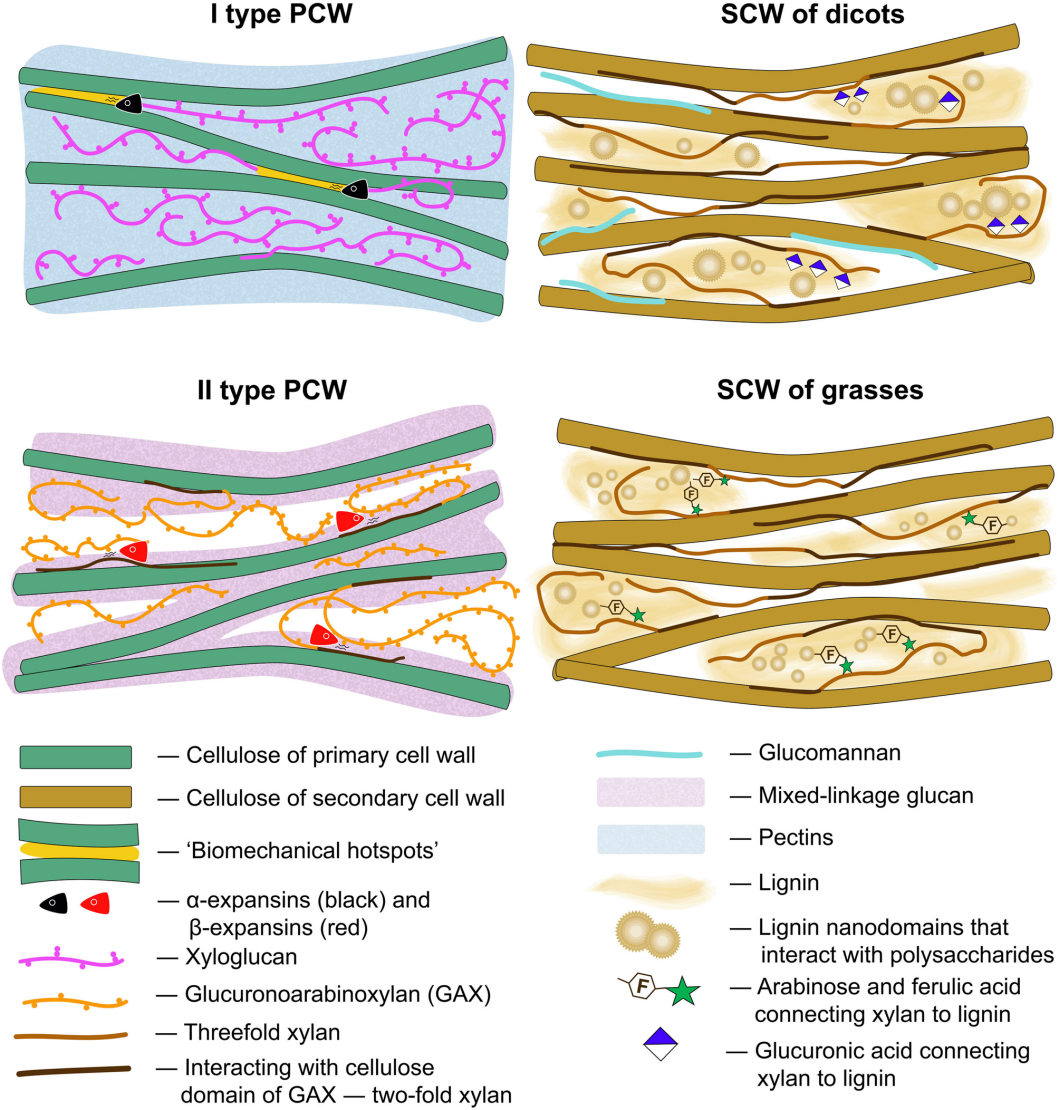
Different types of plant cell wall. Models may not be to scale. Based on (chronological order) Buckeridge et al.^6^, Kozlova et al.^7^, Kiemle et al.^9^, Wang et al.^8^, Simmons et al.^14^, Cosgrove^2^, Kang et al.^13^, Coomey et al.^15^.

The ability to expand or to elongate many times compared to the initial size is a vital property of plant cells. Cells which are capable to grow are surrounded by a thin primary cell wall (PCW). The enlargement of plant cells occurs under the action of turgor pressure and is controlled by the mechanical properties of their cell walls. Mechanical properties, in turn, depend on the cell wall composition and architecture. The mechanisms underlying the growth of plant cells have mainly been studied in dicotyledonous species and non-commelinoid monocots with type I primary cell walls (Fig. 1). Cellulose in the form of microfibrils is present in plant cell walls of all types. Type I cell walls also have pectins and xyloglucans (XyGs) as the basic constituents^1^. Hydrated pectin matrix fills the spaces between cellulose microfibrils. The major part of XyGs also exists between microfibrils in a coiled conformation or interacts with them in an extended conformation. However, minor portion of XyGs is entrapped between cellulose strands^2^. These local interactions of XyGs with cellulose named "biomechanical hotspots" were proposed to form microfibril junctions and integrate them into one load-bearing network^3^. The modification of these junctions by α-expansins enables the irreversible microfibril movements required for cell wall expansion^2^. Alterations in the pectin structure are also considered a potential mechanism regulating wall expansion. Changes in cell wall hydration, the degree of cross-linking or accessibility of individual molecules to degrading enzymes are supposed to be a mechanism underlying the modulation of cell wall mechanics by pectin modifications^4,5^.

The Poaceae family, which includes cereals, is characterized by type II primary cell walls (Fig. 1), where mixed-linkage glucans (MLGs) and glucuronoarabinoxylans (GAXs) predominate, while XyGs and pectins are present at low levels^1^. MLGs, together with low substituted GAXs are thought to cover cellulose, while high substituted GAXs are localized in a space between microfibrils^6–8^. However, in vitro studies have shown that neither arabinoxylan (AX) nor MLG serve as equivalents of either XyG or pectin in interactions with cellulose^9,10^. An α-expansin treatment of grass cell walls does not cause the same loosening response as in type I cell walls^11,12^. β-Expansins, a clade of the expansin family that has substantially evolved in grasses, interact with highly substituted GAXs and rhamnogalacturonans I (RGs-I), but not with cellulose or its contacts with low substituted AXs^8^. These observations discourage direct extrapolations of recent findings in the field of plant cell growth mechanisms obtained from dicots onto grasses.

Many specialized cells, like tracheids, vessels, fibers and sclereids, deposit thickened secondary cell wall (SCW) inside the primary cell wall. This provides additional mechanical strength to plant tissues. Usually, it happens after cessation of expansion growth because solid SCW is not extensible. However, when secondary thickenings have a spiral or annular character of deposing they do not hinder cell elongation. SCWs (Fig. 1) are composed mainly of cellulose, xylans and lignin; the latter is a branched phenolic polymer. Interaction of these three polymers occurs mainly through xylans^13^. The pattern of xylan backbone decoration determines the conformation of this polysaccharide and provides the conditions to interact either with cellulose or with lignin^13,14^. Grasses have a more complex structure of both lignin and xylans^15^. However, the general architecture of SCW is similar in grasses and dicots (Fig. 1).

The importance of the set and structural nuances of cell wall polysaccharides for plant development is getting more and more recognized. However, the molecular details of the processes in elongating cells of grasses remain elusive and need a combination of approaches and adequate model systems for elucidation. The transcriptomic analysis is a powerful tool for identifying genes that modulate biological processes in a living cell. Over the past decade, numerous RNA-Seq and microarray analyses have been conducted on different grass organ and tissue samples to identify key participants in elongation growth. Maize internodes were among the most popular objects of such studies^16–18^. However, the comparison between elongating and non-elongating internodes shifts the focus to genes involved in growth cessation and secondary cell wall formation. Additionally, actively growing intercalary meristems contain vascular tissues; otherwise, the meristematic regions would interrupt the transport continuity and mechanically weaken the stem^19^. Thus, even in its base, the internode represents a mixture of dividing and differentiated cells, which complicates the analysis.

The primary root is a more convenient model system to study elongation growth. Several zones containing cells at different stages of development can be separated from each other based on the distance from the root apex^20^. This unique characteristic was partially employed in the large-scale analysis of transcriptomes^21^ and proteomes^22^ in various parts of the maize root system. Relatively large root fragments that combined several zones were used in these studies, increasing the difficulty of distinguishing the cell division and cell elongation stages, and identifying the important processes occurring at the transition between these stages.

**Figure 2:**
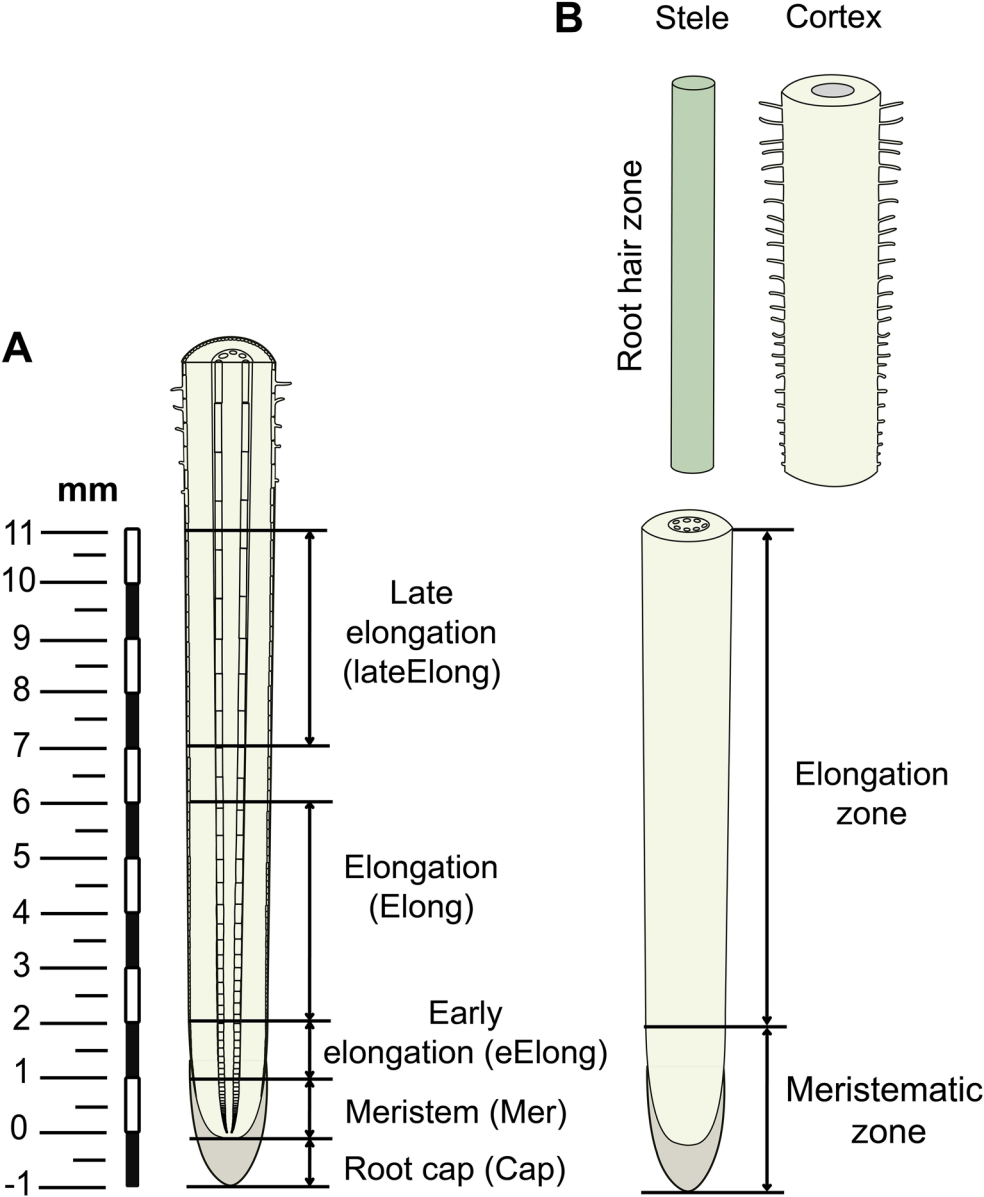
Schematic representation of the collection of samples from maize root in the current study (**A**) and for the proteome analysis reported by Marcon et al.^22^ (**B**).

We have applied an RNA-Seq analysis to five zones in the apical part of maize root (before root hair emergence) to reveal key participants involved in the initiation, realization and cessation of coordinated elongation growth in a plant with type II cell walls (Fig. 2). Clustering and co-expression approaches used for genes encoding numerous glycosyltransferases (GTs) involved in the biosynthesis of cell wall polysaccharides, coupled with extensive immunohistochemical analysis to determine the distribution and dynamics of particular polysaccharide motifs, revealed several patterns of cell wall polysaccharide deposition that corresponded to important stages in cell development.

## Results

Ten individual libraries of mRNAs from two biological replicates of five different samples of maize primary root were analyzed using Illumina sequencing technology. Three hundred thirty million cleaned and filtered 60 bp single-end Illumina reads with quality scores greater than Q30 were used in further analyses. Notably, 87.91% to 89.84% of the reads in each sample were mapped to the reference gene set of the version 4 maize genome, and expression values in units of TGR were calculated. The average value of Pearson’s correlation coefficient for all replicates was greater than 0.95, and a clustering tree of the replicates also indicated the consistency of the data. The annotation of the *Z. mays* B73 AGPv4 (https://ensembl.gramene.org/Zea_mays) contains 44,146 genes, of which 39,324 are defined as protein-coding genes. Across all samples, 26,661 genes were identified, and 26,389 protein-coding genes were expressed with normalized TGR values > 16 at least in one sample. GTs were identified in the genome (B73 RefGen_v4) according to the presence of characteristic Pfam domains in the amino acid coding sequences (Table S1). Two hundred sixty-four genes belonging to 12 GT families and one methyl-transferase family were expressed in maize root. Their expression patterns were analyzed using a clustering analysis, and 6 clusters were identified (Table S1). The phylogenetic analysis of GTs and the comparison with known members of the same GT families in rice and *Arabidopsis* were performed to further characterize the genes and determine the clade of the family (Fig. S1–S10).

### Cellulose synthase superfamily

The biosynthesis of the backbones for several cell wall polysaccharides is mediated by the enzymes encoded by members of the cellulose synthase (CesA) gene superfamily. CesA genes of maize were identified by the presence of PF03552, PF00535, and PF13632 Pfam domains in their protein sequences. The phylogenetic tree was built with known members of CesA superfamily in *Arabidopsis* and rice (Fig. S1). Maize B73 RefGen_v4 contained 53 gene models of putative CesA superfamily genes that, together with *Arabidopsis* and rice genes, were distributed in nine clades. Among the three examined species, the CslB clade was represented only by *Arabidopsis* sequences, while the CslF and CslH clades included only rice and maize genes.

**Figure 3:**
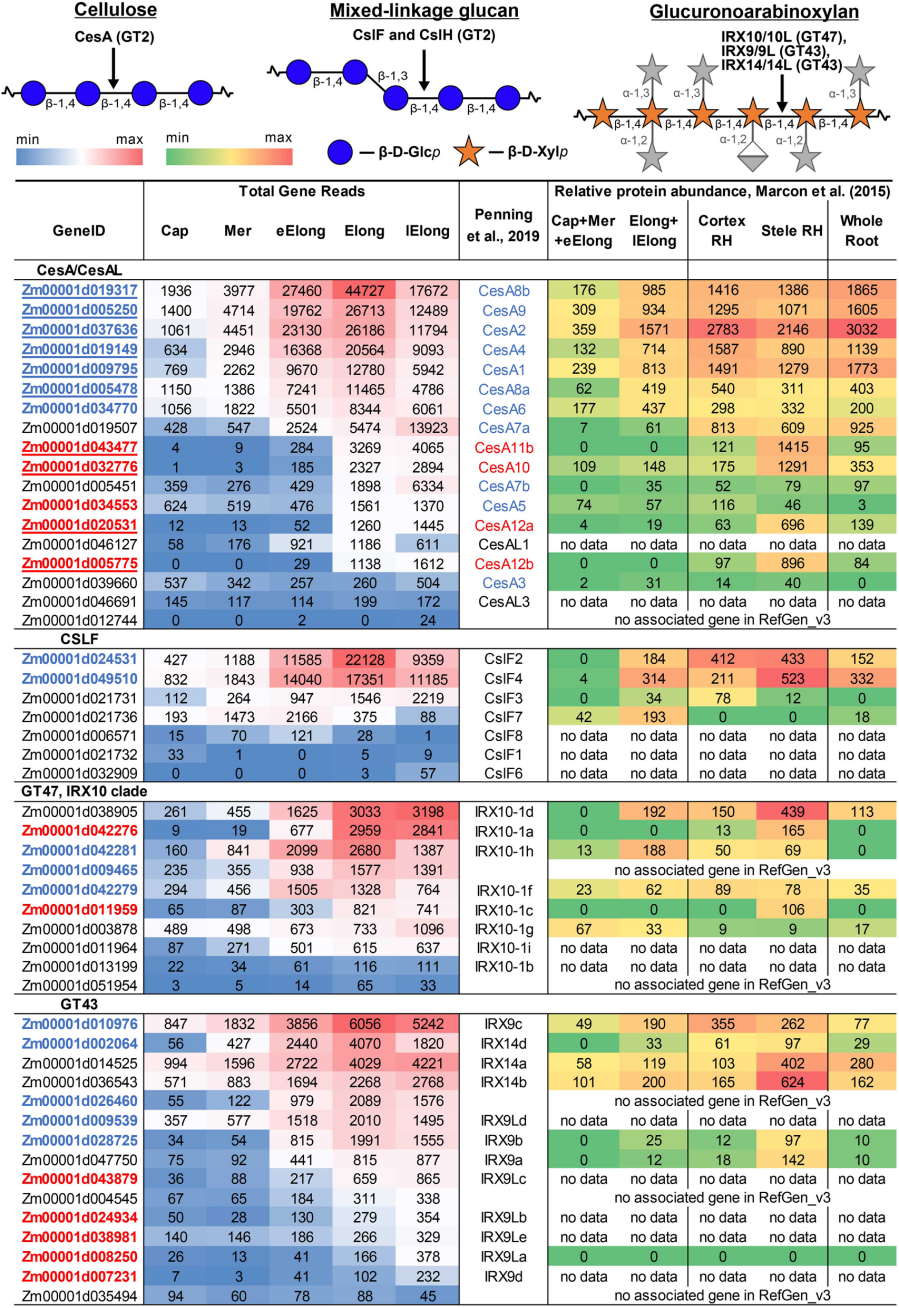
Expression level (TGR, red-blue heat map) and relative protein abundance (averaged and normalized total spectral counts^22^, red-green heat map) of ZmCesA/CesAL, ZmCslFs and genes encoding members of the xylan backbone synthase complex in various zones of maize root. Heat map color coding is applied separately to each gene subgroup. The underlined gene names indicate the baits for co-expression analysis. The genes co-expressed with maize primary cell wall CesAs are labelled in blue, and genes co-expressed with secondary cell wall CesAs are labelled in red. Annotations are based on the study by Penning et al.^18^, and are obtained by matching of the RefGen_v3 and RefGen_v4 gene models. The annotations shown in blue and in red are CesAs assigned to primary and secondary cell wall formation, respectively, by Penning et al.^18^. Cap—root cap, Mer—meristem, eElong—early elongation zone, Elong—zone of active elongation, lElong—zone of late elongation before root hair initiation, and RH—root hair zone. No data, i.e., no corresponding peptides were obtained from any of the studied root samples^22^.

Nineteen maize genes were grouped into the CesA/CesAL clade (Fig. S1). Two recent studies reported 20 members of this clade in maize^18,23^ however, both studies used older versions of the *Zea mays* genome. The new genome assembly associated two gene models, CesA9 (GRMZM2G018241) and CesAL4 (GRMZM2G150404), with the same gene Zm00001d005250. Similarly, two isoforms of CesA11, GRMZM2G037413 and GRMZM2G055795, were merged into one Zm00001d043477 gene. In contrast, Zm00001d012744, which had no associated gene models in previous genome assemblies, entered the CesA/CesAL list as CesA11a according to the phylogenetic analysis (Fig. S2). Seventeen CesA/CesAL genes were expressed in maize root with TGRs greater than 16 at least in one zone (Fig. 3).

**Figure 4:**
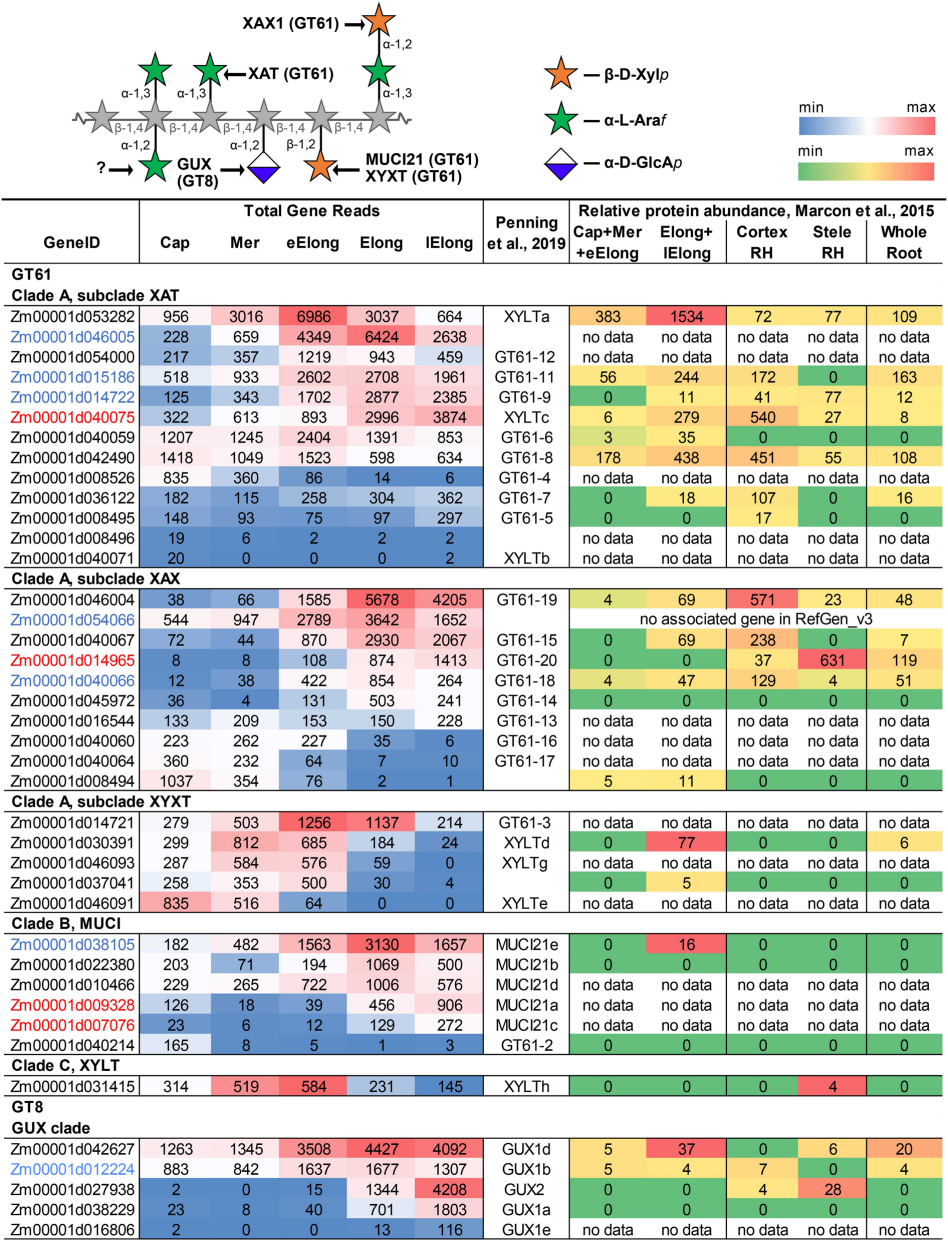
Expression level (TGR, red-blue heat map) of genes potentially involved in GAX backbone decoration in maize root and relative levels of the corresponding proteins (averaged and normalized total spectral counts^22^, red-green heat map. Heat map color coding is applied separately to each gene subgroup. The genes co-expressed with maize primary cell wall CesAs are labelled in blue, and genes co-expressed with secondary cell wall CesAs are labelled in red. Annotations are based on the study by Penning et al.^18^, and are obtained by matching the RefGen_v3 and RefGen_v4 gene models. Cap—root cap, Mer—meristem, eElong—early elongation zone, Elong—zone of active elongation, lElong—zone of late elongation before root hair initiation, and RH—root hair zone. No data, i.e., no corresponding peptides were detected in any of the studied root samples^22^.

**Figure 5:**
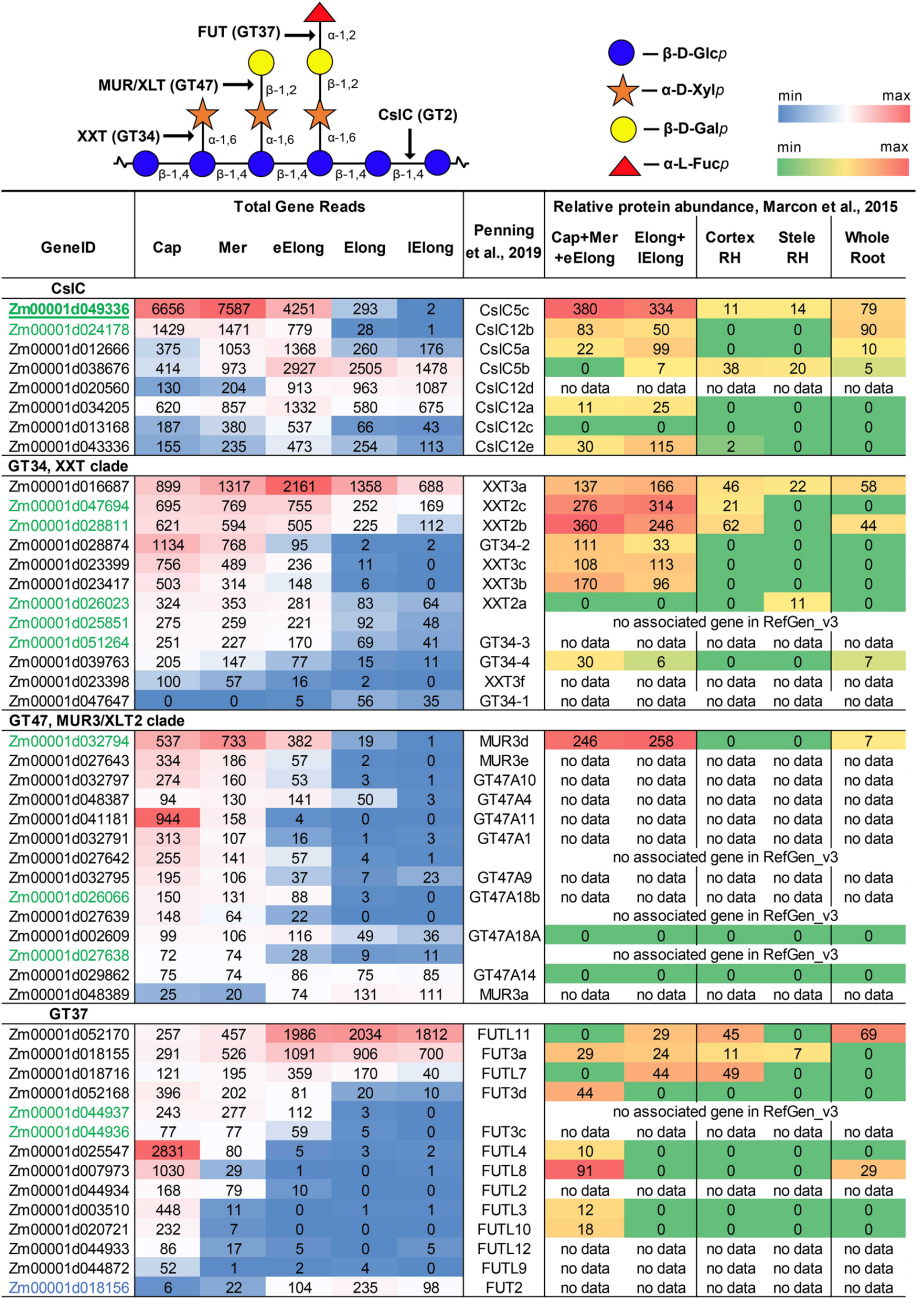
Expression level (TGR, red-blue heat map) and relative protein abundance (averaged and normalized total spectral counts^22^, red-green heat map) of genes potentially involved in XyG synthesis in maize root. Heat map color coding is applied separately to each gene subgroup. The gene co-expressed with maize primary cell wall CesAs is labelled in blue. Green indicates genes co-expressed with ZmCslC5c (underlined) and displaying a correlation coefficient greater than 0.95. Annotations are based on the study by Penning et al.^18^, and are obtained by matching the RefGen_v3 and RefGen_v4 gene models. Cap—root cap, Mer—meristem, eElong—early elongation zone, Elong—zone of active elongation, lElong—zone of late elongation before root hair initiation, and RH—root hair zone. No data, i.e., no corresponding peptides, were detected in any of the studied root samples^22^.

**Figure 6:**
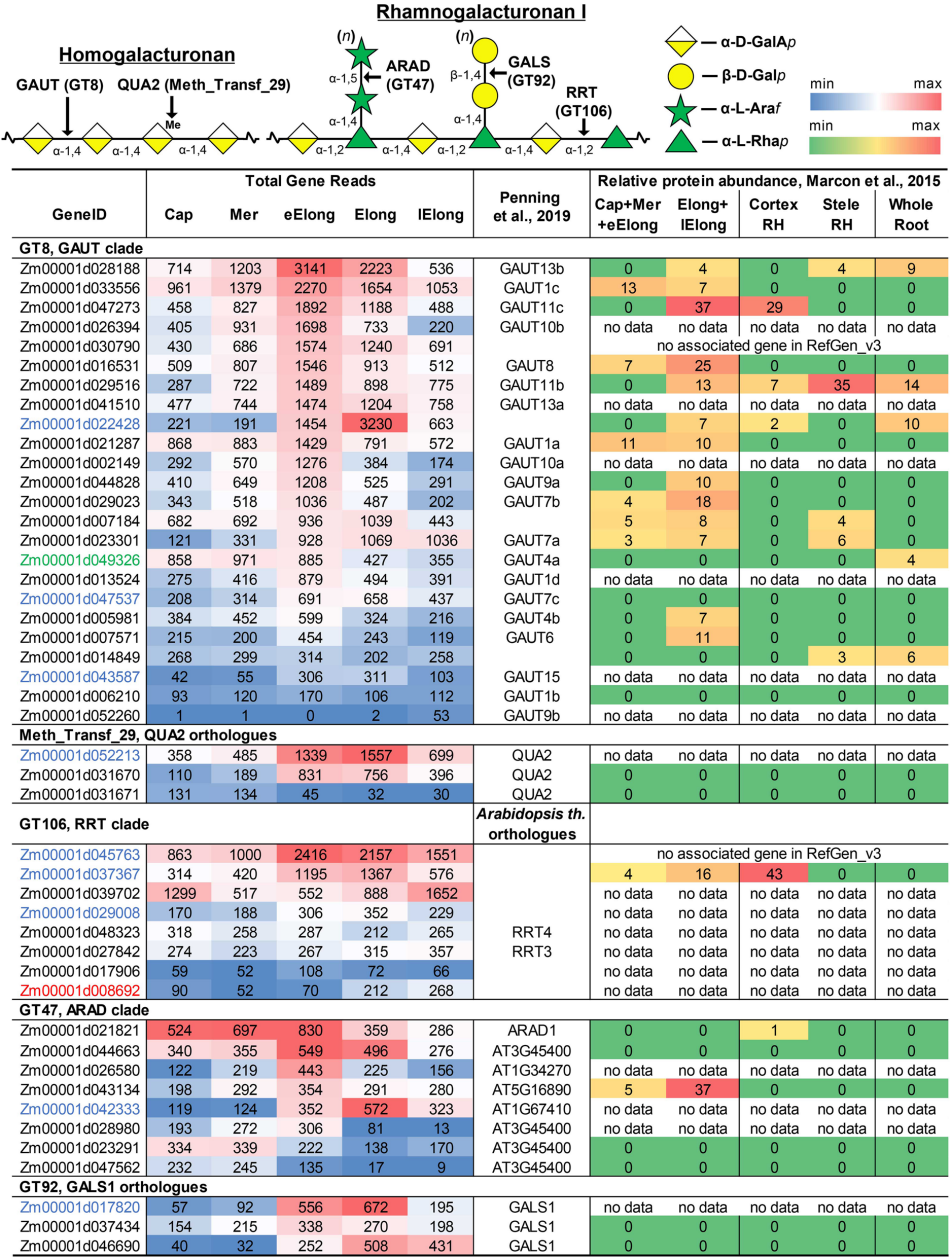
Expression level (TGR, red-blue heat map) and relative protein abundance (averaged and normalized total spectral counts^22^, red-green heat map) of genes potentially involved in HG and RG-I biosynthesis in maize root. Heat map color coding is applied separately to each gene subgroup. The genes co-expressed with maize primary cell wall CesAs are labelled in blue, and genes co-expressed with secondary cell wall CesAs are labelled in red. Green indicates genes co-expressed with ZmCslC5c presenting correlation coefficients greater than 0.95. Annotations are based on the study by Penning et al.^18^, and are obtained by matching the RefGen_v3 and RefGen_v4 gene models Cap—root cap, Mer—meristem, eElong—early elongation zone, Elong—zone of active elongation, lElong—zone of late elongation before root hair initiation, and RH—root hair zone. No data, i.e., no corresponding peptides, were detected in any of the studied root samples^22^.

Penning et al.^18^ proposed that all isoforms of genes ZmCesA1 through ZmCesA9 were involved in primary cell wall synthesis, while genes ZmCesA10 through ZmCesA12 and their isoforms were associated with secondary cell wall biosynthesis. ZmCesA1, 2, 4, 6, 8a/b and 9 displayed similar expression profiles along the root length. Transcripts of these genes were relatively abundant in the meristem zone. Four- to five-fold up-regulation was characteristic of these genes in the early elongation zone, with further increase in the elongation zone and two-fold down-regulation at the late elongation stage. According to the proteomic study performed by Marcon et al.^22^, corresponding proteins were present in the meristematic region of maize seedling primary root and accumulated during elongation. Both stele and cortex tissues in the root hair region of young maize root were characterized by high levels of these cellulose synthases^22^. These features of the transcription and translation of particular cellulose synthase genes probably reflect the high demand for new cell wall material by rapidly elongating cells. The six mentioned cellulose synthases were co-expressed with each other, with correlation coefficients greater than 0.95. They were used as the bait genes for the co-expression analysis to reveal other GTs governed by the same regulatory pattern (Table S1, net_pcw column, Figs. 3–6).

The ZmCesA10-12 genes were expressed at low levels in the root cap, meristem and early elongation zone. Significant increases in the levels of their transcripts occurred in the elongation zone, with further increases in the TGR values in the late elongation zone. High levels of the corresponding proteins were detected in the stele of the root hair region of maize root^22^. A virtual absence of certain transcripts and proteins at the earlier stages of cell development, transcriptional up-regulation in the elongation zone that peaked at the stage of late elongation, and the high levels of proteins in the stele of the root hair region corresponded to the development of the vascular system and secondary cell wall thickening in maize root. Four isoforms of maize CesAs (10, 11, and 12a/b) were co-expressed, with correlation coefficients greater than 0.95, and were selected as bait genes to build the co-expression network related to secondary cell wall biosynthesis (Table S1, net_scw column; Figs. 3–6).

Members of the cellulose synthase-like H, F and J clades of the CesA superfamily mediate MLG synthesis in grasses and heterologous expression systems^24^. According to the phylogenetic tree, seven sequences of maize genes were grouped with rice CslFs, and one with rice CslHs genes (Fig. S1). No representatives of CslJ subfamily were found.

Two maize orthologues of the rice CslF6 gene (Zm00001d024531 and Zm00001d049510) exhibited the highest TGRs in the active elongation zone and were co-expressed ZmCesAs, which are predicted to be involved in primary cell wall formation (Fig. 3 and Table S2). Another member of the CslF clade (Zm00001d021736, the orthologue of rice and barley CslF3) was up-regulated in the meristem and early elongation zone. This GT was recently shown to possess novel activity, mediating the synthesis of glucoxylan^25^. Protein products of this gene were detected in maize root segments before, but not after, root hair initiation^22^.

### Glucuronoarabinoxylan (GAX) synthesis

GAXs are the major noncellulosic polysaccharides present in the type II primary walls. The synthesis of its β-1,4-xylan backbone is mediated by a complex of three proteins. Two of these proteins (IRX9 and IRX14) represent the GT43 family (Fig. S3), and the other (IRX10) belongs to a separate clade of the GT47 family (Fig. S4). Xylosyl-transferase activity in vitro has only been observed for IRX10^26,27^. IRX9 and IRX14 are believed to function as structural components of the xylan synthase complex with no catalytic activity *per se*^26^.

Maize RefGen_v4 contains 51 genes encoding proteins with the predicted PF03016 Pfam domain, which is the signature of the GT47 family. Four of them were shorter than 270 amino acids and were excluded from further analysis. The phylogenetic tree with *Arabidopsis* GT47 members was built to identify maize orthologues of AtIRX10 and AtIRX10L (Fig. S4). The IRX10 clade of GT47 included ten paralogous genes of maize. All were expressed in maize root, with the highest TGR values observed in the zones of active or late elongation (Fig. 3). Three isoforms of ZmIRX10 (Zm00001d042281, Zm00001d009465, and Zm00001d042279) were co-expressed with primary cell wall CesAs, and two others (Zm00001d042276 and Zm00001d011959) were co-expressed with secondary cell wall CesAs (Fig. 3). The GT43 family is represented in the maize genome by 15 members encoding proteins containing the PF03360 domain (Fig. S3). All these genes were expressed (Fig. 3). Ten belonged to either primary or secondary cell wall-related co-expression networks.

The GAX backbone can be decorated with arabinose, glucuronic acid, xylose or oligosaccharide chains. The attachment of arabinosyl and xylosyl residues to the GAX molecule is mediated by GTs of the GT61 family^28,29^, while GT8 members are required for the glucuronosyl substitution of the xylan backbone^30^.

The GT61 family has been subdivided into several clades with specific characterized members (Fig. S5). Clade A contains maize orthologues for rice OsXAT2 (Zm00001d053282) and OsXAT3 (Zm00001d046005), as well as wheat TaXAT1 (Zm00001d036122). XATs are xylan α-1,3-arabinofyranosyl-transferases^28^. The majority of genes in the XAT subgroup of the maize GT61 family were expressed with the maximum TGR values in the early elongation or elongation zones (Fig. 4).

One additional characterized member of GT61 clade A is OsXAX1. The enzyme encoded by this gene adds β-1,2-xylose to the α-1,3-arabinose side-chain of the xylan backbone^29^. Three maize genes are present on the branch of GT61 tree that contains OsXAX1. Zm00001d046004 had highest TGR value among all genes belonging to this subclade. Two other genes on the same branch (Zm00001d054066 and Zm00001d014965) were co-expressed with primary or secondary cell wall-related CesAs, respectively (Fig. 4).

Another member of GT61 clade A, XYXT1, functions in rice as a xylosyl-transferase attaching β-1,2-xylosyl side-chains onto the xylan backbone^31^. The maize orthologue of OsXYXT1 (Zm00001d014721) had highest TGR values in the early elongation and elongation zones, and was significantly down-regulated in the late elongation zone of maize root (Fig. 4).

Clade B of the GT61 tree contains AtMUCI21 and six genes of maize that were expressed in the primary root. AtMUCI21 was characterized as a β-1,2-xylosyl-transferase, a xylan branching enzyme of *Arabidopsis* seed mucilage^32^. Three maize members of the GT61 clade B were predominantly expressed in the elongation zone, while two others were expressed in the late elongation zone of maize root.

The enzymes known to mediate the attachment of α-1,2-GlcA side-chains onto the xylan backbone in dicots^33,34^ represent a separate clade of the GT8 family (Fig. S6). Five of these genes were expressed in maize primary root, with the maximum of TGR values in either the active elongation or late elongation stages.

Thus, two different sets of GTs, both of which are sufficient for the production of highly substituted xylans, are expressed in maize primary root. Genes and proteins of these enzymes appear along the root length in a manner corresponding to primary and secondary cell wall biosynthesis.

### Xyloglucan (XyG) synthesis

XyGs consist of a backbone of 1,4-linked β-d-glucosyl residues, some of which are substituted at O(6) with an α-d-xylosyl residue. Further substitution of xylose with galactose or fucosyl-galactose may occur in cell walls of Poales^35,36^.

The formation of the XyG backbone is catalyzed by members of the CslC clade of the GT2 family^37^. CslC4 of *Arabidopsis* induced XyG backbone synthesis in heterologous systems^38^. The maize genome contains eight members of the CslC clade (Fig. S1); all eight genes were expressed in maize root (Fig. 5). Two orthologues of the *Arabidopsis* CslC5 gene (Zm00001d049336 and Zm00001d024178) were expressed at high levels, with maximum TGR values observed in the root cap and meristem (Fig. 5). A greater than 25-fold decrease in transcript abundance was observed in the zone of active elongation. Proteins encoded by these genes were expressed at the highest levels in the meristematic region of maize root^22^. Zm00001d049336 is also the orthologue of OsCslC3, which is predicted to function as a XyG backbone synthase in rice^36^. This gene was chosen as the bait in the co-expression analysis to identify other participants in XyG synthesis in maize (Fig. 5 and Table S1, xyg_net column).

The XyG backbone is decorated by xylose residues. In *Arabidopsis*, the attachment of xylose is catalyzed by members of the GT34 family XXT1, 2, 4 and 5^39,40^. The maize genome possesses 18 genes containing the Pfam domain (PF05637) characteristic of GT34. Twelve representatives of XXT clade (Fig. S7) were expressed (Fig. 5). The maximum levels of the transcripts of almost all GT34 members were observed in the root cap or meristem zone of maize root. Expression decreased at later stages of cell development. Five of these genes were co-expressed with ZmCslC5c (Fig. 5 and Table S1). Proteins of the GT34 family were mainly detected in the elongating part of maize root, and rarely in the root hair region.

Xylose residues attached to the XyG backbone may be further substituted at the O(2) position with galactose. Two GTs of the GT47 family, MUR3 and XLT2, are predicted to be involved in XyG galactosylation in *Arabidopsis*^41^. Representatives of the GT47 family possess various activities, and the family is divided into several clades (Fig. S4). The MUR3/XLT2 clade contained 14 genes expressed in maize root. The vast majority of these genes were expressed with the highest TGR values in root cap and meristem zones. A recent study of *Sorghum* revealed two proteins functioning as the XyG galactosyl-transferases^42^. The Zm00001d032794 gene is an orthologue of one of these genes in maize. This gene and two other members of the MUR3/XLT2 clade were co-expressed with ZmCslC5c (Fig. 5).

Fucose-containing side-chains of XyG are barely detectable in Poaceae species^35^. However, fucogalactoxyloglucans were recently identified in young tissues of rice^36^. XyG fucosylation is catalyzed by members of the GT37 family^43^. Nineteen genes belonging to this family were recognized in the RefGen_v4 maize genome, according to the presence of the PF03254 Pfam domain. Fourteen of these genes were expressed in analyzed root zones, and among them, four orthologues of AtFUT1 (Zm00001d020721, Zm00001d003510, Zm00001d044933, and Zm00001d044872) known to be involved in fucosylation of XyGs in *Arabidopsis*^44^ and one orthologue of rice OsFUT1 (Zm00001d052168) were detected. All five presented the highest TGR values in the root cap zone, and corresponding proteins were observed only in the meristematic part of maize root (Fig. 5).

### Pectin synthesis

Pectins are minor components of type II cell walls, comprising only 2–10% of the dry mass^45^. Homogalacturonan (HG) usually is the most abundant pectic polysaccharide. It consists of an α-1,4-linked d-galacturonic acid (GalA) backbone, which is synthesized by HG galacturonosyl-transferases (GAUTs)^46–48^. GAUTs belong to the GT8 family (PF01501), which also includes xylan α-glucuronyl-transferases (GUXs) and glucoside α-1,3-galactosyltransferases (GATLs) participating in GAX and AGP synthesis, respectively (Fig. S6).

Forty-seven members of the GT8 family were identified in the fourth version of the maize genome, although three of these genes are too short to encode active enzymes. Twenty-four of these genes belong to the GAUT clade (Fig. S6). The vast majority of GAUTs exhibited maximum TGR values in the early elongation or elongation zone (Fig. 6). Corresponding proteins were not detected at high levels, but were mainly observed in the elongating part of the root in the study by Marcon and colleagues^22^.

HG is synthesized in a highly methyl-esterified form and is postulated to lose ester groups during the elongation growth of cells^5^. The attachment of methyl groups is mediated by methyl-transferases belonging to the Methyl_Transf_29 family possessing the characteristic PF03141 Pfam domain. Fifty-seven genes encoding proteins with this domain were identified in maize RefGen_v4, 41 of which were expressed in maize primary root. AtQUA2 is characterized on the protein level as pectin-methyl-transferase in *Arabidopsis*^49,50^. Two orthologues of AtQUA2 in maize (Zm00001d052213 Zm00001d031670) were expressed with high TGR values; however, no corresponding proteins were detected in a proteomic study^22^ (Fig. 6).

The backbone of rhamnogalacturonan I (RG-I) is composed of repeating diglycoside 2-α-l-Rha*p*-1,4-α-d-Gal*p*A-1 units. Recently, members of GT106 (RRT clade), which were previously annotated as fucosyl-transferases, were shown to catalyze rhamnose attachment to the RG-I backbone acceptor^51^. The RRT clade is represented by eight genes in the maize genome (Fig. S8). All of these genes were expressed in the primary root. Only the protein encoded by Zm00001d037367 was detected^22^. This gene and two other members of this clade belonged to the co-expression network of CesAs genes responsible for primary cell wall formation.

The RG-I backbone can be substituted at the (O)2 position of rhamnosyl residues. Side-chains usually are represented by β-1,4-d-Gal*p* or α-1,5-l-Ara*f* oligosaccharides. The synthesis of β-1,4-galactan in *Arabidopsis* is mediated by the GT92 family member AtGALS1^52^. Three orthologues of this gene are present in the *Zea mays* genome. All of these genes were expressed in the apical part of maize root. Other members of the GT92 family exhibited low similarity with GALS1. No corresponding proteins were detected^22^. However, two of the three maize GALS1 orthologues were co-expressed with primary cell wall CesAs (Fig. 6).

Another type of side-chains for RG-I is α-1,5-l-arabinan, the synthesis of which is catalyzed by AtARAD1 in *Arabidopsis*^53^. This enzyme belongs to the GT47 family (clade C) (Fig. S4). Eight members of this clade were expressed in the maize primary root. The orthologue of AtARAD1 (Zm00001d021821) presented higher TGR values in the root cap, meristem and early elongation zone (Fig. 6). However, corresponding proteins were detected only in the cortex of the root hair region^22^.

### Immunohistochemistry

The presence of transcripts and proteins of GTs essential for the synthesis of a particular polysaccharide does not indicate that this polymer is indeed produced. We performed immunohistochemical analysis with a set of monoclonal antibodies to trace the dynamics of cell wall polysaccharides during the elongation growth of maize root.

**Figure 7:**
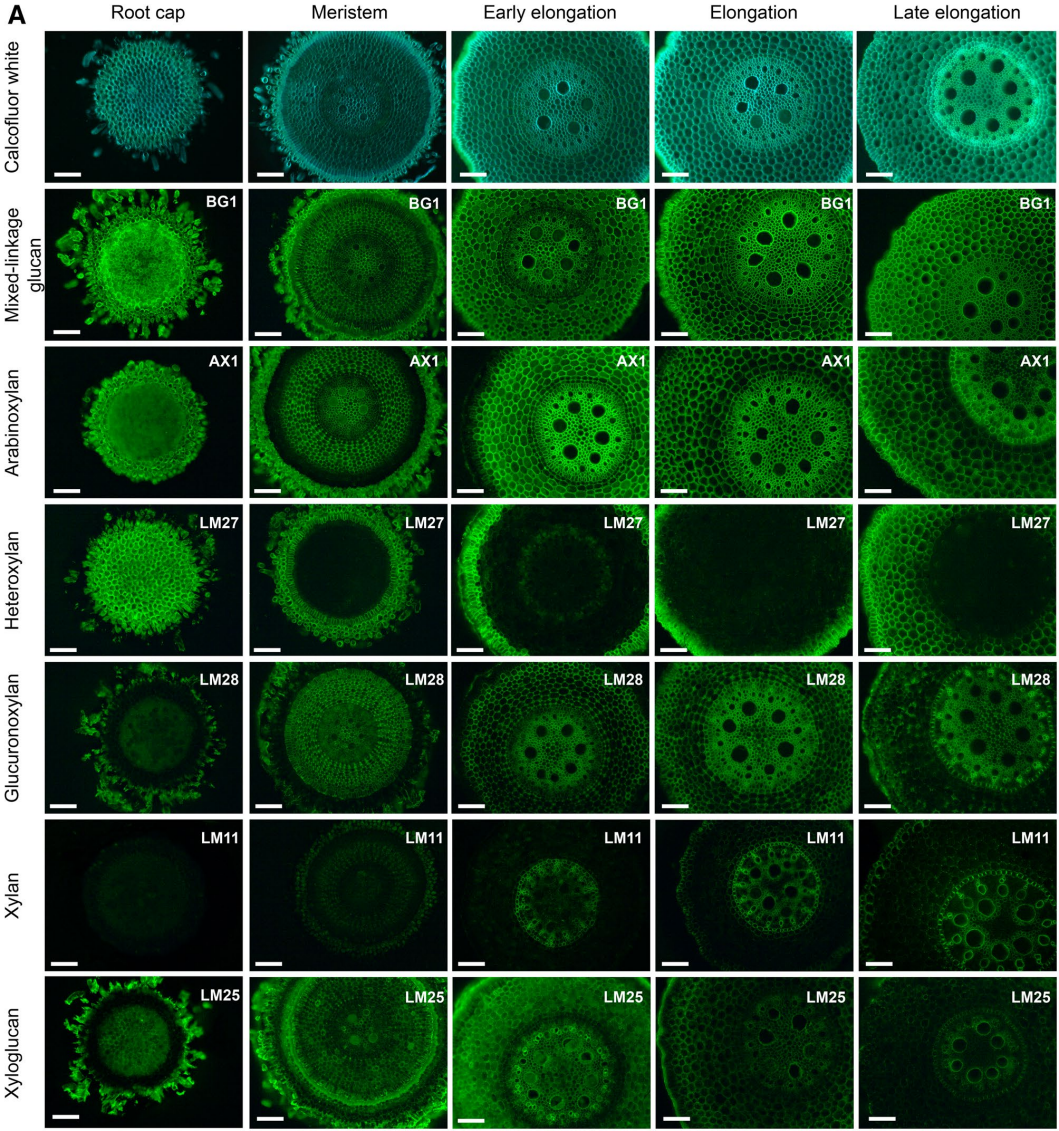

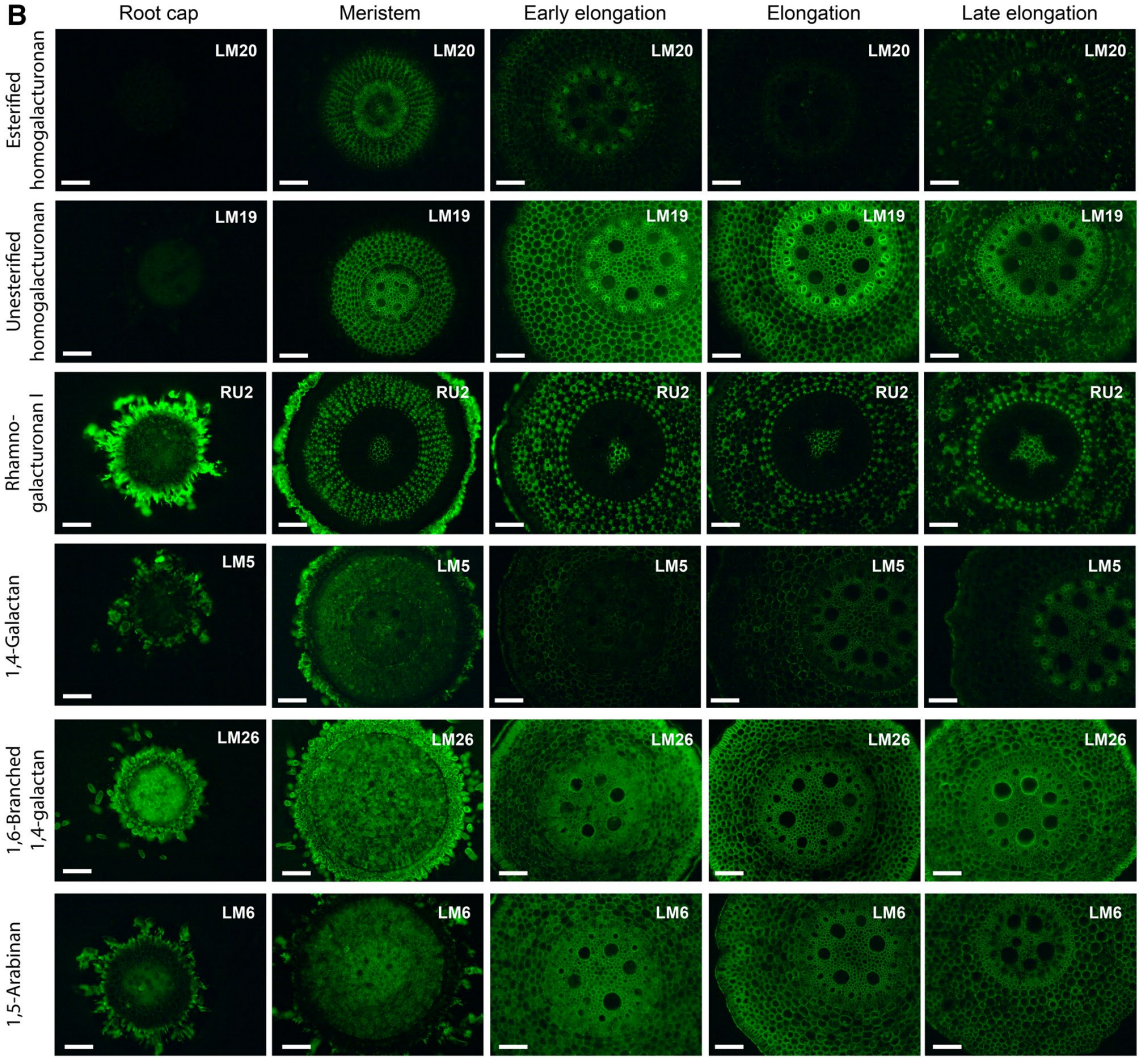
Fluorescence micrographs of maize root sections stained with Calcofluor White and immunolabelled by BG1 (mixed-linkage glucan), LM25 (galactoxyloglucan), AX1 (arabinoxylan), LM27 (grass heteroxylan), LM28 (glucuronoxylan), LM11 (low-substituted xylan), LM20 (esterified homogalacturonan), LM19 (un-esterified homogalacturonan), RU2 (rhamnogalacturonan I backbone), LM5 (1,4-β-galactan), LM6 (1,5-α-arabinan), and LM26 (1,6-branched 1,4-β-galactan) antibodies. Bar—100 μm. No fluorescence was detected in negative control samples (primary antibodies were omitted) under the observation conditions.

Calcofluor White stained cell walls in maize root evenly in the meristem, early elongation and elongation zones (Fig. 7, Calcofluor White). Yellow coloration appeared in the vascular parenchyma in the late elongation zone, indicating its lignification. Brighter staining of the vascular ring corresponded to secondary cell wall thickening (Fig. 7).

BG1 and AX1 antibodies, which bind MLGs^54^ and AXs^55^, respectively, labelled maize cell walls in all investigated zones (Fig. 7, BG1 and AX1). However, their labelling was weaker in meristem region. The rhizodermis was only labelled by the AX1 antibody in the late elongation zone. The LM27 antibody recognizing grass heteroxylans^56^ did not label meristematic cells. At the stage of early elongation, it bound the rhizodermis and outer cell layer of the cortex. A similar pattern was observed in the elongation zone. Labelling was increased centripetally in the late elongation zone and observed in several layers of the cortex (Fig. 7, LM27). The LM28 antibody specific for glucuronoxylans^56^ labelled all tissues except rhizodermis. The preference for binding to stele tissues was observed in the early elongation, elongation and late elongation zones (Fig. 7, LM28). The LM11 antibody is often used to detect secondary cell wall xylans. It was raised against xylooligosaccharides^57^ and probably requires a less substituted backbone fragment in contrast to other anti-GAX probes used in the current study. The LM11 antibody labelled vascular tissues in the elongation and late elongation zones. Brighter labelling was also observed in the rhizodermis in the late elongation zone (Fig. 7, LM11).

The epitope of LM25, the antibody specific for galactoxyloglucans^58^, was detected at relatively high levels in the meristematic region of maize root and root cap cells, and in the slime produced by these cells. The labelling intensity increased in the early elongation zone and then decreased in the elongation and late elongation zones. However, the root slime in these zones still bound the antibody (Fig. 7, LM25). A similar pattern of labelling was observed with another xyloglucan-specific antibody, LM15 (data not shown).

LM19 and LM20 antibodies are used to detect unesterified and methyl-esterified HGs, respectively^59^. LM20 epitopes were present only in the meristematic region, and the labelling became weaker in subsequent zones. In contrast, LM19 labelling became more intense in the early elongation zone. Rhizodermis or root slime did not possess the epitopes for the LM19 antibody. Elongation and late elongation zones were characterized by stronger labelling with the LM19 antibody in the root stele. A higher level of these epitopes was observed in the vascular parenchyma and phloem/protoxylem ring in the late elongation zone (Fig. 7, LM20 and LM19).

The RU2 antibody recognizes the backbone of RG-I^60^. The root cap cells and root slime were labelled by this antibody. Cell walls in cell corners of middle cortex and pith were strongly labelled throughout root development (Fig. 7, RU2). Three antibodies specific for RG-I side-chains were also used. 1,4-Galactans (LM5^61^) were detected in root cap cells or slime and root meristematic tissues, excluding the rhizodermis. The early elongation zone possessed lower levels of epitopes for the LM5 antibody, while a gradual increase in the labelling intensity was observed in stele tissues in the elongation and late elongation zones. Phloem cells were enriched in LM5 epitopes (Fig. 7, LM5). The recently developed LM26 antibody marks β-1,4-galactan branched at the O(6) position with another galactosyl residue^62^. LM26 labelling was much stronger than LM5 labelling (Fig. 7, LM26). All tissues analyzed at all of the developmental stages possessed epitopes for the LM26 antibody. The staining with the LM6 antibody (α-1,5-arabinan^63^, was also intense. The antibody was distributed evenly in all tissues and in all zones of maize root (Fig. 7, LM6).

### Major patterns of cell wall formation

**Figure 8:**
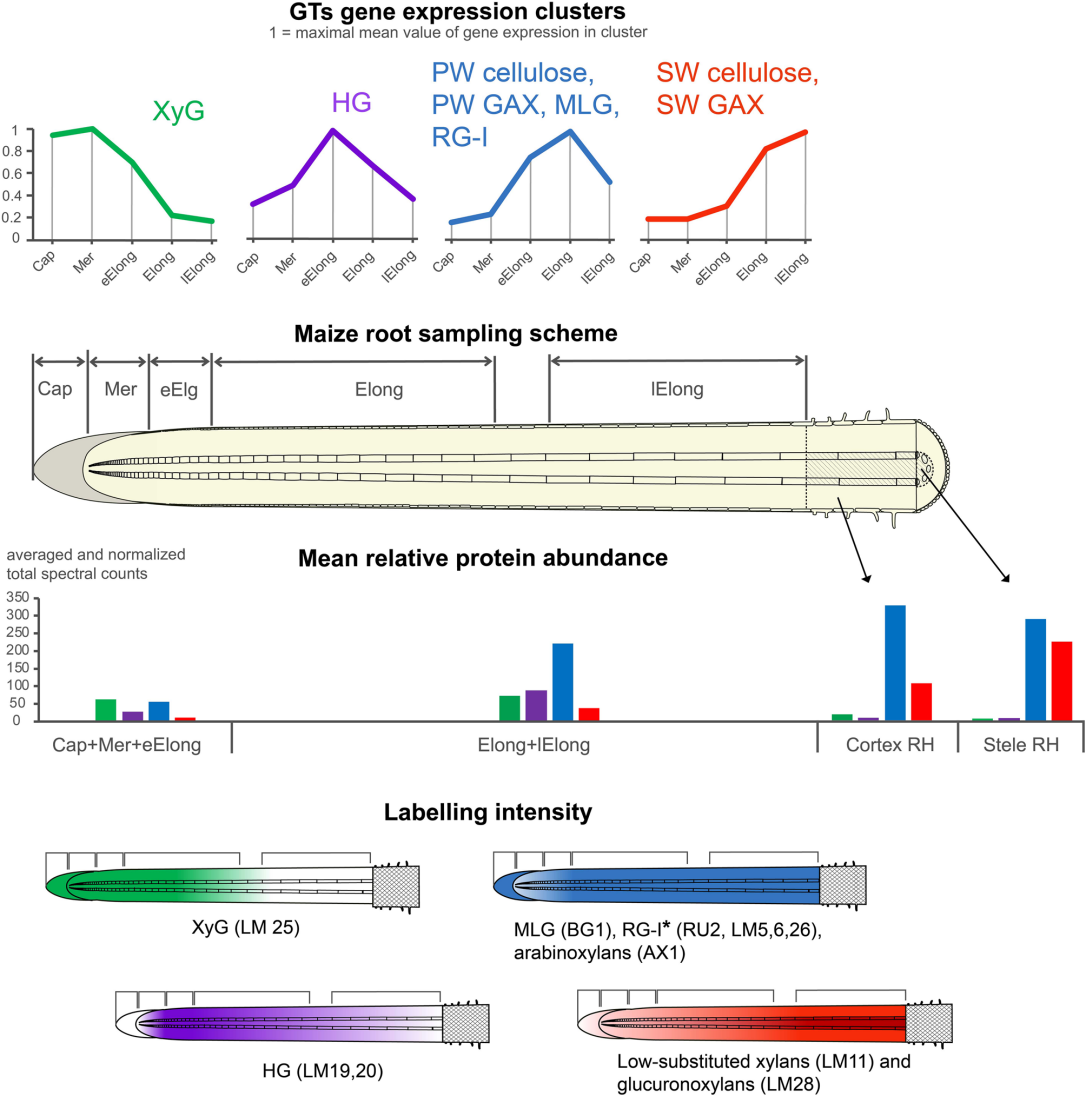
Four major clusters of GT expression in maize root. Expression profiles represent normalized and averaged TGR values for each cluster. Polysaccharides were assigned to a particular cluster based on the proportion of corresponding GTs included in the cluster. Protein levels are shown on a diagram as means for all members of a particular cluster. Labelling intensity is shown as color gradient where white corresponds to weak labelling. XyG—xyloglucan, HG—homogalacturonan, PW—primary cell wall, GAX—glucuronoarabinoxylan, MLG—mixed-linkage glucan, RG-I—rhamnogalacturonan-I, SW—secondary cell wall. Cap—Root cap, Mer—meristem, eElong—early elongation zone, Elong—elongation zone, and lElong—late elongation zone, Cortex RH and Stele RH—cortex and stele in root hair region, respectively. *RG-I related antibodies had equal intensity of labelling in all studied root zones.

Transcriptomic data for all GTs recognized in the maize genome were subjected to a cluster analysis. Six clusters were revealed, four of which were the most populated (Fig. 8 and Table S1). Cluster 1 included genes expressed at high levels in the root cap and meristem zone and at lower levels in subsequent zones of maize root. It was enriched with numerous GTs potentially involved in XyG biosynthesis. According to the data reported by Marcon et al.^22^, corresponding proteins were observed in maize root before, but rarely after, root hair initiation (Fig. 8). XyG epitopes were present in the cell walls of the meristem and early elongation zone (Fig. 7, LM25).

Members of another cluster displayed peak expression in the early elongation zone. This cluster was enriched with representatives of GT8 (GAUT clade), GT31 and GT2 (ZmCslF3 and ZmCslF7), putative mediators of HG backbone, arabino-galactan protein and glucoxylan synthesis, respectively. Proteins encoded by these genes were present in the apical part and elongation zone of maize root, but not in the root hair region^22^ (Fig. 8). Esterified HGs disappeared from root sections at the beginning of elongation (Fig. 7, LM20), while unesterified pectins persisted in sections throughout development (Fig. 7, LM19). Polysaccharide synthases belonging to this cluster may have specific roles in the transition to elongation.

Transcripts of many GTs present in the meristem zone exhibited a substantial increase in expression in the early elongation zone, peaked in the active elongation zone, and subsequently decreased in the late elongation zone (Fig. 8). These GTs included cellulose synthase isoforms related to PCW deposition, MLG synthases, some GAX backbone and side-chain synthases, and RG-I backbone and side-chain synthases. Many of these genes were combined into one co-expression network when PCW CesAs were used as baits (Table S1). A proteomic study^22^ revealed the accumulation of the corresponding proteins between the apical part of the root and root hair region. In meristem region this amount was comparable with the level of XyG-related proteins. At further stages of development these proteins became the most abundant among all studied GTs (Fig. 8). Antibodies specific for polysaccharides synthesized by GTs of this cluster (BG1 for MLG; AX1 for GAX; and RU2, LM5, LM6, and LM26 for RG-I) bound the cell walls of maize root the most actively (Fig. 7). However, the labelling by BG1 and AX1 was weaker in meristem. RG-I specific probes were distributed uniformly.

Low expression in the root cap, meristem and early elongation zone and increased expression in the elongation and late elongation zones were characteristics of the fourth major pattern of GT expression (Fig. 8). CesAs responsible for secondary cell wall formation and many GAX-related synthases were pooled into this cluster and into one co-expression network. Corresponding proteins were specifically abundant in the stele of the root hair region of maize root^22^ (Fig. 8). These features of transcription and translation correlate with the development of the vascular system and SCW thickening in maize root, as visualized using Calcofluor staining and LM11, and LM28 labelling (Fig. 7).

## Discussion

The type II primary cell wall is considered the characteristic feature of grasses^1^. The content of XyG and pectins—the major constituents of type I primary cell walls—is usually reported to be less than 10% in grass cell walls^17,64,65^. However, both the set of expressed genes for various GTs and distribution of epitopes for numerous antibodies reveal the working machinery required to synthesize XyG and pectins (Fig. 8). The GTs implicated in XyG synthesis were actively expressed in the root cap and meristem (Fig. 5 and 8). Distinct presence of XyG was confirmed using immunolabelling (Fig. 7, LM25). Thus, the active synthesis of XyG indeed occurs at early stages of grass cell development. We suggest that XyG may be present at high levels in the walls of meristematic cells. Its content in coleoptiles and internodes typically used for investigations may be low since no or few dividing cells are present in these plant parts. The high XyG level may be necessary to form the required density of “biomechanical hotspots” to initiate elongation in a manner similar to dicots.

In elongation and late elongation zones of maize root the mRNA level of genes involved in XyG biosynthesis drops down, XyG labelling intensity decreases (Fig. 8). However, recent study of the maize coleoptile glycome and proteome revealed the unexpected abundance of XyGs in the Golgi apparatus that contrasted relatively low content in cell wall^65^. Together with the earlier report of XyG internalization from the cell wall and transport to the cytoplasm in maize root meristem^66^, these data indicate the possibility of XyG recycling in elongating cells. The circulation of XyGs that were initially synthesized at the stage of division may provide each new layer of the cell wall with a sufficient amount of XyG-based “biomechanical hotspots” if they are necessary for elongation growth. However, supposing XyG-based “biomechanical hotspots” indeed mediate an elongation growth of grass cells, it is unclear why α-expansins have no substantial effect on the creep of plants with the type II cell walls^67^. The clue may be the fact that β-expansin clade did not replace α-expansins in grass genomes but was developed additionally^67^. As an assumption, the coordinative action of these two expansin families may be necessary to induce elongation.

HG is another component typical for type I cell walls which presence may affect the architecture of grass cell walls. The expression of many genes encoding HG backbone synthases peaked at the transition to elongation (Fig. 6 and 8). Intensive labelling by HG-recognizing antibodies was observed in meristem and early elongation zones (Fig. 7, LM19, LM20). The increased content of pectins in the meristematic region of maize root as compared to elongation zone was detected by a higher proportion of uronic acids in corresponding segments^68,69^. We have observed a decrease in the level of HG methylation at the beginning of elongation growth (Fig. 6). In dicots, the reduction in pectin methylation correlates with increased cell wall extensibility^70–72^. Methyl-esterified HG disappeared from maize root sections at early elongation stage, while un-esterified HG persisted for a longer time (Fig. 7, LM19, LM20). To our knowledge, this study is the first to show a change in the level of pectin methylation during the elongation growth of cereal root.

Along with the level of methyl-esterification of HGs, the type and relative abundance of RG-I side-chains may modulate cell wall mechanics^62,73,74^. Basically, a higher level of side-chains and higher branching degree correspond to a more elastic cell wall. This increase in elasticity is proposed to be the consequence of pectins high water-holding capacity and ability to provide the properties of fluid-saturated poro-elastic material to cell walls^4^.

RG-I related genes were co-expressed with primary cell wall cellulose synthases and MLG synthases, with the peak expression observed at the stage of active elongation. Immunolabelling with the corresponding antibodies was more or less uniform along the root length (Fig. 7, RU-2, LM5, LM26, and LM6).

Based on these findings, the machinery necessary to generate the set of components for the type I primary cell wall operates in growing maize root. Thus, the basic mechanisms of cell wall surface enlargement involving XyGs, HGs and RGs-I may be valid for plant species with both types of primary cell walls.

GAX and MLG are the renowned constituents of the primary cell wall of grasses^1^. What privileges do they provide to the plant organism, if the basic mechanism for cell enlargement may be established in grasses in a similar manner to dicots?

The maximum expression of transcripts encoding MLG synthases (ZmCslFs) was observed in the active elongation stage of root development (Fig. 8), and these synthases were co-expressed with primary cell wall-related cellulose synthases (Fig. 3 and Table S1). Together with long-lasting idea of MLG importance for cell expansion^7,64,75–77^, this polymer is currently often considered a short-term storage of glucose independent of starch metabolism^78^. Any impediment to MLG turnover may decrease the energy supply, what explains some previously reported findings, such as the increased turnover of MLG during rapid cell growth^79,80^ or inhibition of auxin-induced growth by antibodies that bind MLG or MLG-degrading enzymes, preventing the hydrolysis of this polysaccharide^81,82^. Thus, MLG may combine the functions of the reserve and a filler of the space between cellulose microfibrils during their movement away from each other in the course of cell elongation.

Representative members of GT families involved in GAX biosynthesis are expressed differently (Figs. 3, 4,8). Some of these genes display transcriptional and translational profiles similar to primary cell wall cellulose synthases, while the others are co-expressed with secondary cell wall cellulose synthases (Fig. 8 and Table S1). Homologous genes displaying altered expression according to two these profiles were detected in GT families engaged in the biosynthesis of both the backbone and side-chains of GAX. Thus, two separate gene sets may be responsible for the biosynthesis of GAX for primary and secondary cell wall deposition in maize root (Figs. 3 and 4). Further diversity of GAX molecules in cell walls of maize root is evidenced by the accumulation of LM27 epitope specifically in rhizodermal and cortical cells (Fig. 7). This epitope is not yet characterized, but is presumed to be a complex substitution of grass heteroxylans^56^. Complex GAX are thought to be important for the formation of hemicellulose:lignin complexes, cessation of growth, and in defense mechanisms^83^.

G(A)Xs of the maize root primary cell wall and secondary cell walls of dicots have specific domains^84,85^ (Fig. 1). Different parts of these molecules have different patterns of backbone substitution and different abilities to interact with cellulose^86^. Therefore, one molecule may serve as both bonding agent and spacer for cellulose microfibrills, as it is suggested in the models of grass cell wall^7, 13^.

Thus, MLG and GAX presence may provide increased diversity, domain structure, and/or additional functions; however, these properties do not directly explain their importance in elongation growth per se. This importance is strongly suggested by the dynamics of MLGs and GAXs accumulation. Both these polymers exist in cell walls of maize root already in meristem, but account just a few percent of cell wall weight^85,87^. Cell walls of quiescent center of maize root are totally free of MLG, as was demonstrated by immunocytochemistry^87^.The proportion of MLGs and GAXs within cell walls increases gradually from meristem to late elongation zone; at active elongation stage these polysaccharides characteristic of type II cell walls become dominating. Besides, one of the well-documented modifications of type II primary cell walls during elongation growth is the decrease in GAX substitution with arabinose^64,77,80^, which may alter the domain proportion and modify the GAX:cellulose interactions^7^. The dynamics of arabinofuranosidase gene transcription and corresponding activity in the crude extracts gradually increases from the meristem to late elongation zones^88^, indicating the involvement of GAX modification in some steps of elongation process. And still, there is no sufficient information to answer the question on the privileges obtained by grasses due to the presence of GAX and MLG.

Polysaccharide ensemble of cell walls in maize root is dynamically changed in the course of elongation growth. At earlier stages of development, components characteristic of type I primary cell walls are actively deposited. Type II specific polysaccharides exist in cell walls of maize root already in the meristem; however, they become dominating only when the elongation rate reaches a maximum. Growth cessation is coupled to the diversification of GAX molecules in cell walls of different tissues. Mechanisms of elongation growth in grasses are still far from understanding. However, new participants of this process revealed by a transcriptomic approach should now be taken into account.

## Materials and methods

### Plant material and sample preparation

Investigations were conducted with the roots of 4-day-old maize seedlings (*Zea mays* L., cv. Mashuk (Niva Tatarstana, Kazan, Russia)) grown in the dark at 27 °C. The primary root was subdivided into the zones according to the following pattern: root cap, meristem (0–1 mm), early elongation (1–2 mm), elongation (2–6 mm), and late elongation (7–11 mm) as was described in Kozlova et al.^7^ and Kozlova et al.^89^ (Fig. 2). The 7–11 mm root zone was renamed from post-elongation to late elongation zone in this study.

### RNA extraction and sequencing

Root zones pooled from 30 plants (an average) were collected separately into plastic tubes with liquid nitrogen and stored at −80 °C. Total RNA was isolated from plant samples using the Trizol extraction method combined with RNeasy Plant Mini Kit (Qiagen) according to the manufacturer’s instructions. Residual DNA was eliminated by treating the samples with DNAse I using the DNA-free kit (Ambion). The RNA quantity and quality were confirmed spectrophotometrically with a NanoDrop ND-1000 spectrophotometer (Thermo Fisher Scientific) and using 1% agarose gel electrophoresis. For whole-transcriptome sequencing, cDNA libraries from the total RNA of root segments were prepared with a TruSeq Sample Prep Kit (Illumina) according to the manufacturer’s instruction. Sequencing was performed using an Illumina HiSeq 2500 instrument (Illumina) with single-end 60 bp reads. Data in the form of raw reads and sample preparation descriptions were deposited at NCBI Sequence Read Archive (SRA) and are available at BioProject ID PRJNA639682. All samples were analyzed using RNA-Seq analysis in two independent biological replicates.

### Processing and analysis of the RNA-Seq data

Processing and Analysis of the RNA-Seq Data were as described before by Gorshkov et al.^90^. Adapter removal, quality trimming, and the removal of contaminating sequences, such as rRNAs, tRNAs, snRNAs and snoRNAs, found in the RNACentral DB^91^ were performed using the BBDuk utility of BBToolsv37.02 [BBTools, https://jgi.doe.gov/data-and-tools/bbtools/bb-tools-user-guide/bbduk-guide/ (accessed on 21 January 2019)]. Clean reads for each sample were mapped onto the maize genome sequence using HISAT2 v2.1.083^92^ with the default parameters. The assembly and annotation of a reference genome of maize B73 RefGen_v4 were downloaded from Gramene (https://ensembl.gramene.org/Zea_mays/Info/Index). The read count for each gene was quantified using StringTie v2.0 software^93^ with the default settings. For each gene, total gene reads (TGRs) were determined as the number of all reads that mapped to this gene.

The R (https://www.R-project.org/;95) package DESeq2 v.1.14.1^95^ was used to perform the differential expression analysis of mRNAs from all samples using the TGR counts generated for each sample, as described above. The DESeq estimateSizeFactors and estimateDispersions functions (with the default options) were used to obtain normalization factors for each sample and to normalize the TGR counts. Genes with an average normalized TGR of > 16 in at least in one sample were considered as expressed, according to the recommendations of the sequencing quality control project^96^. The resulting dataset consisted of 26,661 genes that were used for the differential expression analysis.

### Identification of genes coding enzymes involved in cell wall polysaccharide biosynthesis

Predicted full-length protein sequences of different glycosyltransferase (GT) families and methyltransferases were recognized according to presence of characteristic domains and obtained from the Ensembl plants database (https://plants.ensembl.org/index.html;98) for maize, *Triticum aestivum,* and *Sorghum bicolor* genes and for one rice gene. The Phytozome v12.1 database (https://phytozome.jgi.doe.gov/pz/portal.html;99) was used to obtain *Arabidopsis thaliana* and rice protein sequences. The canonical transcripts were used. Genes of different GT families were recognized by the presence of the following domains: PF03552, PF00535, PF13632—GT2, PF01501—GT8, PF00777—GT29, PF01762—GT31, PF055637—GT34, PF03254—GT37, PF03360—GT43, PF03016—GT47, PF02364, PF14288—GT48, PF04577—GT61, PF01697—GT92, and PF10250—GT106. Methyltransferases belonging to 29th family were recognized by the presence of a PF03141 domain. Genes that were too short or possessed additional domains were excluded from the analysis.

### Phylogenetic analysis

The retrieved maize, *Arabidopsis*, and rice (wheat and *Sorghum* in some cases) amino acid sequences were subjected to multiple alignment using the web-based services Clustal Omega (https://www.ebi.ac.uk/Tools/msa/clustalo/,100) and Muscle (https://www.ebi.ac.uk/Tools/msa/muscle/,100). The satisfactory alignments were chosen and subjected to a maximum likelihood phylogenetic analysis which was performed using IQTREE1.6.9 software^100^. The best-fit model of sequence evolution was selected using Akaike Information Criterion (AIC) and Bayesian Information Criterion (BIC) implemented in ModelFinder (IQTREE1.6.9)^101^ and the ultrafast bootstrap branch support with 10,000 replicates^102^ was used to construct each dendrogram. Trees were visualized using the web-based service iTOL 5.3 (https://itol.embl.de/;104).

### Clustering and co-expression analyses

The gene co-expression was analyzed with the Comparative Co-Expression Network Construction and Visualization tool (CoExpNetViz) using the Pearson correlation coefficient^104^. The averaged normalized TGR values (TGR > 16 in at least one sample) were used as the input file. Genes were considered co-expressed if their correlation was greater than the 95th percentile of the correlation distribution between samples of genes per gene expression matrix. The network was visualized using Cytoscape version 3.7.2^105^. The normalized values of expression were used to cluster recognized GTs using functions from different R packages (cluster, factoextra, and dendextend) (https://www.R-project.org/;95). The Euclidean distance and the Pearson correlation coefficients were used to group the samples and to scale and group the genes, respectively. The Ward.D2 method was used in both cases.

### Proteomic data processing

Tissue-specific expression of proteins in primary maize root has been determined by Marcon et al.^22^. Normalized and averaged data are available at Maize Genetics and Genomics Database (https://www.maizegdb.org/) and as supplemental material in Walley et al.^106^. Data conversion from B73_RefGen_v3 to B73_RefGen_v4 coordinates was accomplished in R software environment (https://www.R-project.org/;95) by using maize.v3TOv4.geneIDhistory.txt file, which was downloaded from ftp://ftp.gramene.org/. Data on GT expression were separated by gene numbers and are presented in Table S1.

### Immunohistochemistry

Dissected maize roots were incubated for 1 h with 4% (w/v) paraformaldehyde prepared in 0.2 M phosphate-buffered saline (PBS, pH 7.2). Root fragments were embedded in 3% (w/v) low melting point agarose. Cross Sects. (50 µm thick) were prepared using a Leica VT 1000S (Leica Biosystems) vibratome (blade speed—0.65 mm s^−1^, blade frequency—70 Hz for the meristem, early elongation and elongation zones; blade speed—0.225 mm s^−1^, blade frequency—100 Hz for late elongation zone). Immunohistochemical analysis of cell wall polymers was performed using BG1 (Australian Biosupplies Pty Ltd.), AX1, RU2 (INRA), LM5, LM6, LM11, LM19, LM20, LM25, LM26, LM27, and LM28 (Leeds University) antibodies. For immunolocalization, the sections were (1) blocked with 0.2 M PBS containing 2% (w/v) bovine serum albumin (BSA) for 30 min at room temperature, (2) incubated with one of the primary monoclonal antibodies diluted 1:5 (LM5, LM6, LM11, LM19, LM20, LM25, LM26, LM27, and LM28), 1:10 (BG1) or 1:3 (AX1 and RU2) for 1.5 h at room temperature then washed three times with PBS. (3) Next, the sections were incubated with secondary anti-rat (LM5, LM6, LM11, LM19, LM20, LM25, LM26, LM27, and LM28) or anti-mouse (BG1, AX1, and RU2) IgG linked to fluorescein isothiocyanate (FITC) diluted 1:100 in PBS for 1 h at room temperature in the dark. The primary antibody treatment was omitted for the negative controls. After incubations with the antibodies, the sections were washed four times with PBS and twice with water. For the visualization of the tissue structure, sections were incubated in Calcofluor White 1:100 solution in PBS. Sections were observed using a Leica DM1000 epifluorescence microscope (Leica Biosystems) fitted with a mercury lamp. Sections were observed under epifluorescence settings with the excitation filter BP 480/40 nm and barrier filter BP527/30 nm for FITC-conjugated antibodies, and excitation filter 355–425 nm and barrier filter 470 nm for Calcoflour. All analyses were performed using at least three biological replicates. Antibodies used in the present study are listed in Table S2.

## Supplementary information

Supplementary file1

Supplementary file2

Supplementary file3

## Data Availability

All materials, data, and associated protocols are promptly available to readers without undue qualifications.
