# Supplementary material for: Elongating maize root: zone-specific combinations of polysaccharides from type I and type II primary cell walls
Source: Sci Rep. 2020 Jul 2;10:10956. doi: 10.1038/s41598-020-67782-0 (PMC7331734; doi:10.1038/s41598-020-67782-0)
Supplement: Supplementary file 1 — Supplementary file1 [file 41598_2020_67782_MOESM1_ESM.docx]

**Elongating maize root: zone-specific combinations of polysaccharides from type I and type II primary cell walls**

Scientific Reports

Liudmila V. Kozlova*, Alsu R. Nazipova, Oleg V. Gorshkov, Anna A. Petrova, and Tatyana A. Gorshkova

Kazan Institute of Biochemistry and Biophysics, FRC Kazan Scientific Center of RAS, Laboratory of Plant Cell Growth Mechanisms, Kazan, Russian Federation

* corresponding author: kozlova@kibb.knc.ru


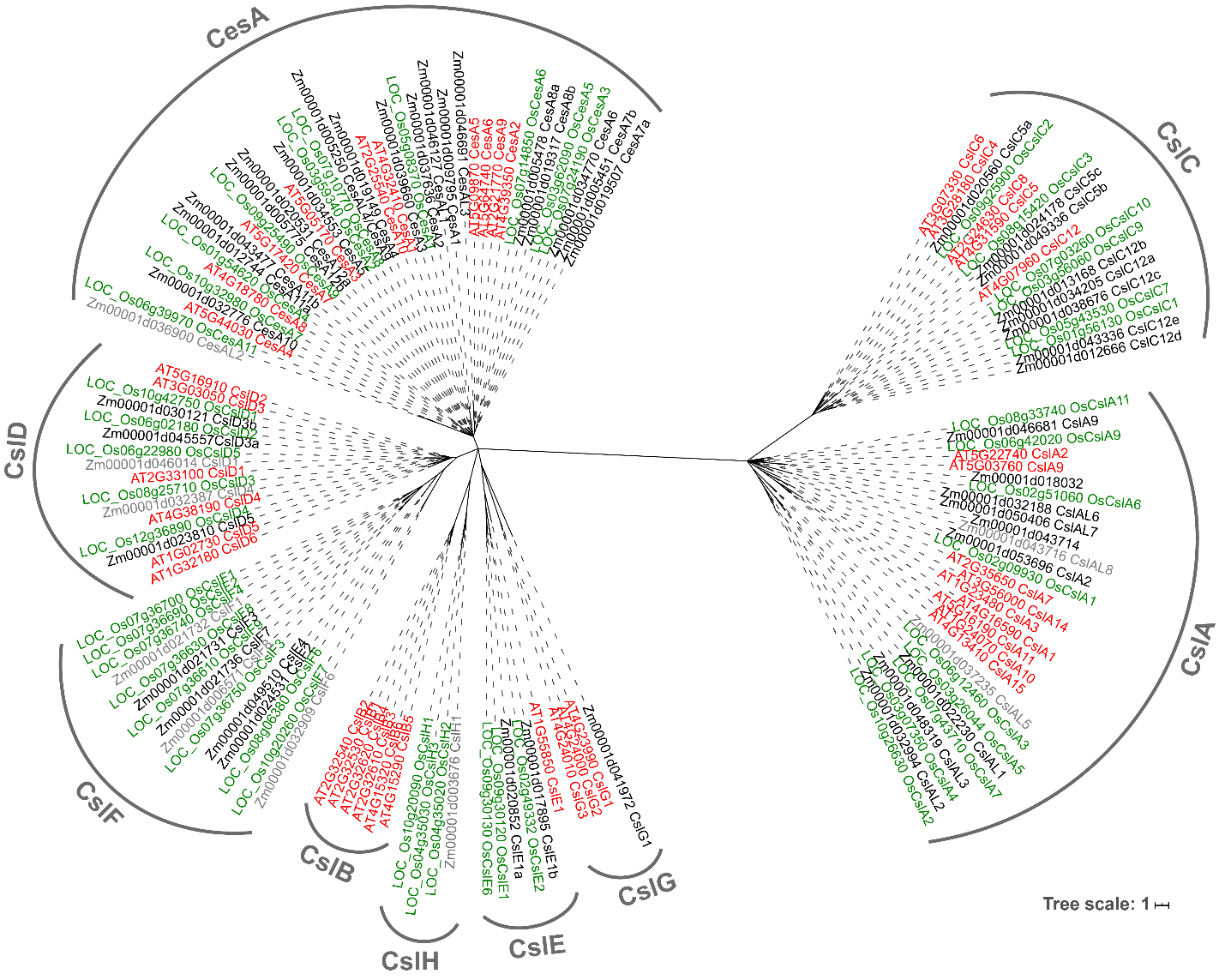


**Fig. S1** Dendrogram of glycosyltransferase (GT) 2 family genes of maize, *Arabidopsis* and rice. Genes labelled in black, red and green correspond to maize, *Arabidopsis* and rice respectively. Grey color indicates maize genes that were not expressed in maize primary root. Maize gene names were added basing on the study by Penning et al. (2019)^1^. *Arabidopsis* genes were named according to Richmond and Somerville (2000)^2^. Rice genes were named according to Wang et al. (2010)^3^. Branches with low confidence ultrafast bootstrap support (<95%) were deleted.


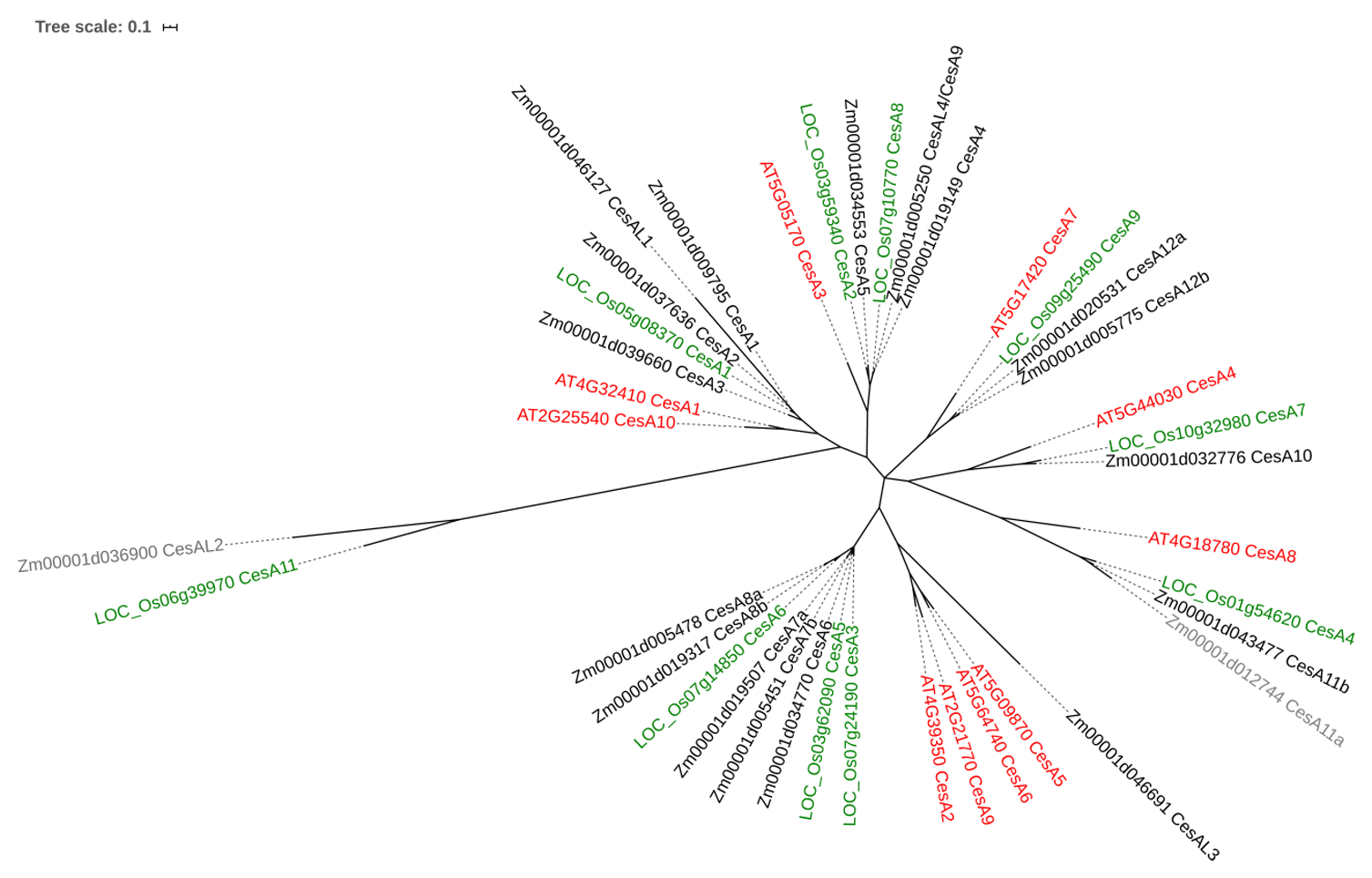


**Fig. S2** Dendrogram of cellulose synthase (CesA) genes of maize, *Arabidopsis*, and rice. Genes labeled by black, red and green correspond to maize, *Arabidopsis* and rice respectively. Grey color indicates maize genes that were not expressed in maize primary root. Maize gene names were added basing on the study by Penning et al. (2019)^1^. *Arabidopsis* genes were named according to Richmond and Somerville (2000)^2^. Rice genes were named according to Wang et al. (2010)^3^. Branches with low confidence ultrafast bootstrap support (<95%) were deleted.


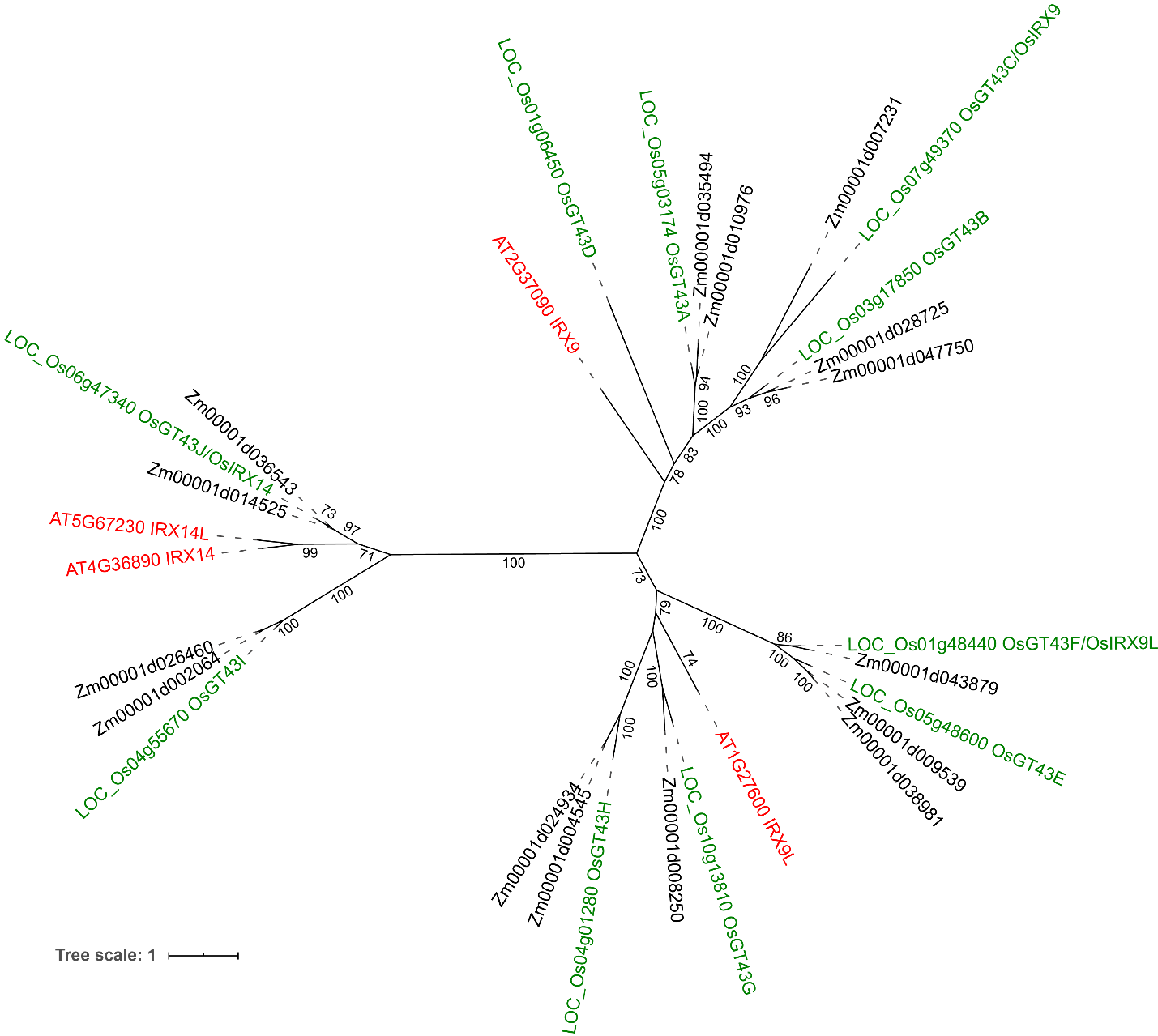


**Fig. S3** Dendrogram of plant GT43 family genes. Genes labeled by black, red and green correspond to maize, *Arabidopsis* and rice respectively. Genes names are based on Chiniquy et al. (2013)^4^ and Lee et al. (2014)^5^. Numbers indicate ultrafast bootstrap branch support for some branches.


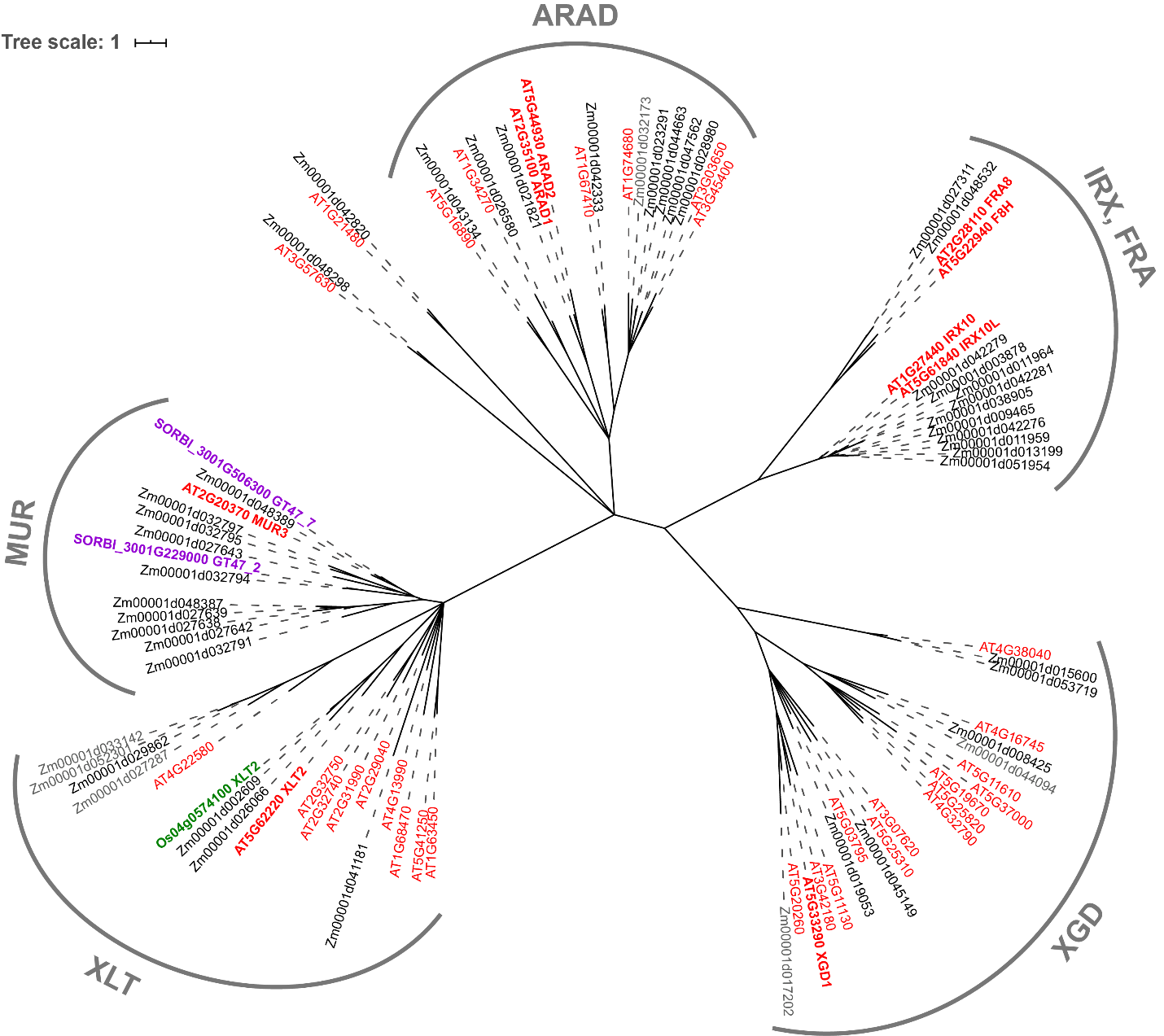


**Fig. S4** Dendrogram of plant GT47 genes. Names of genes labeled by black and red colors corresponds to maize and *Arabidopsis* genes respectively. Two characterized *Sorghum bicolor*^6^ and *Oryza sativa*^7^ GT47 members labelled by purple and green color respectively. Genes encoding characterized proteins are in bold. Grey color indicates maize genes that were not expressed in maize primary root. Branches with low confidence branch support (< 95 percent) were deleted.


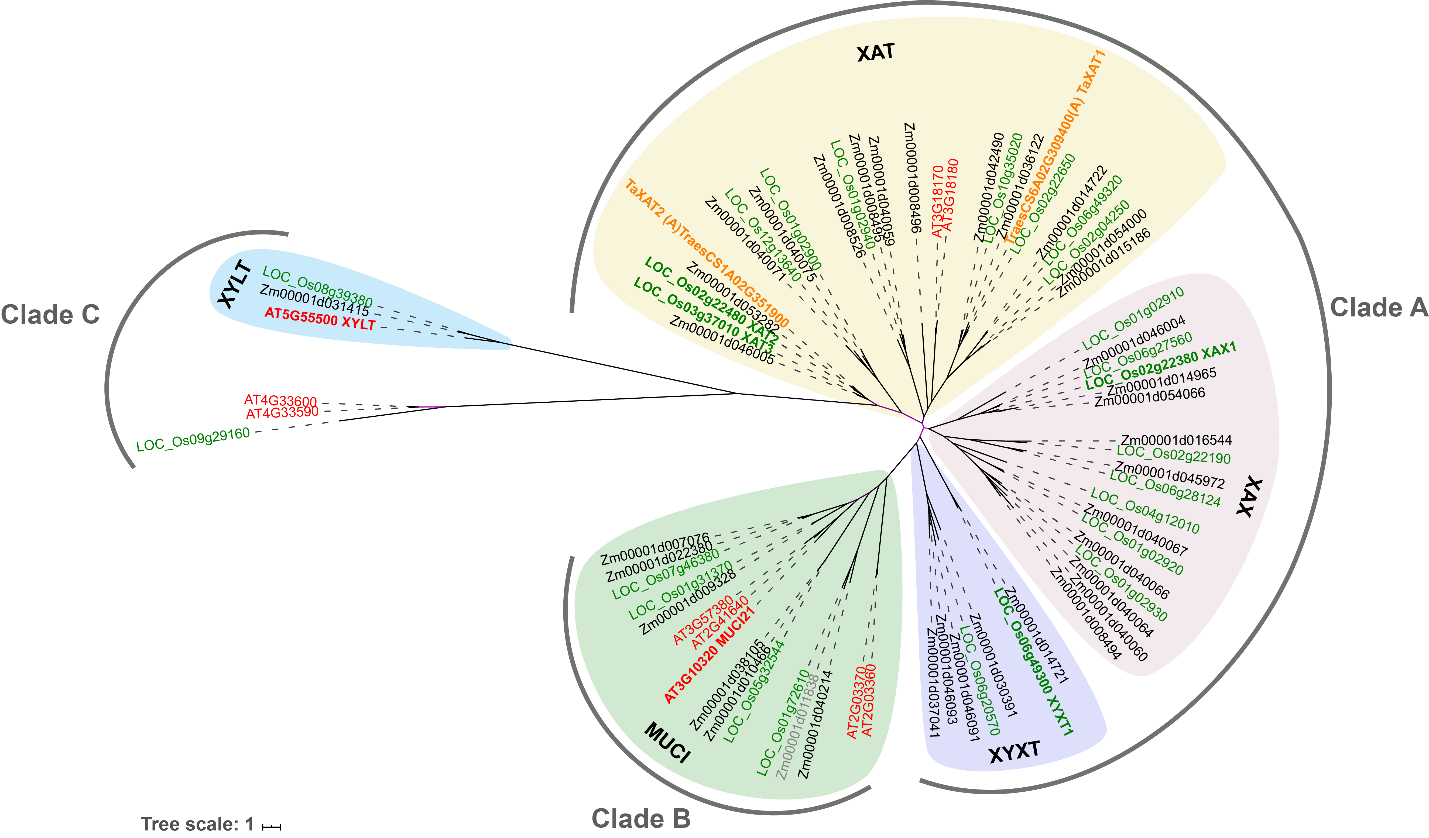


**Fig. S5** Dendrogram of plant GT61 genes. Names of genes labeled by black, red, green and orange correspond to maize, *Arabidopsis*, rice and wheat genes respectively. Grey color indicates maize genes that were not expressed in maize primary root. Names of genes encoding characterized proteins are in bold. Clades are named according to Anders et al. (2012)^8^. Clades C and A are separated from each other at low confidence branch support. Thus, the tree representation differs from Anders et al. (2012)^8^ phylogenetic tree. The division of Clade A to three subclades is conditionally because of low branch support values. Clade C is divided to two subclades and one of them shown by blue color and it have one characterized Arabidopsis XYLT gene. Branch ultrafast bootstrap support shown by range from purple color to black that means range from 40 to 100 percent respectively.


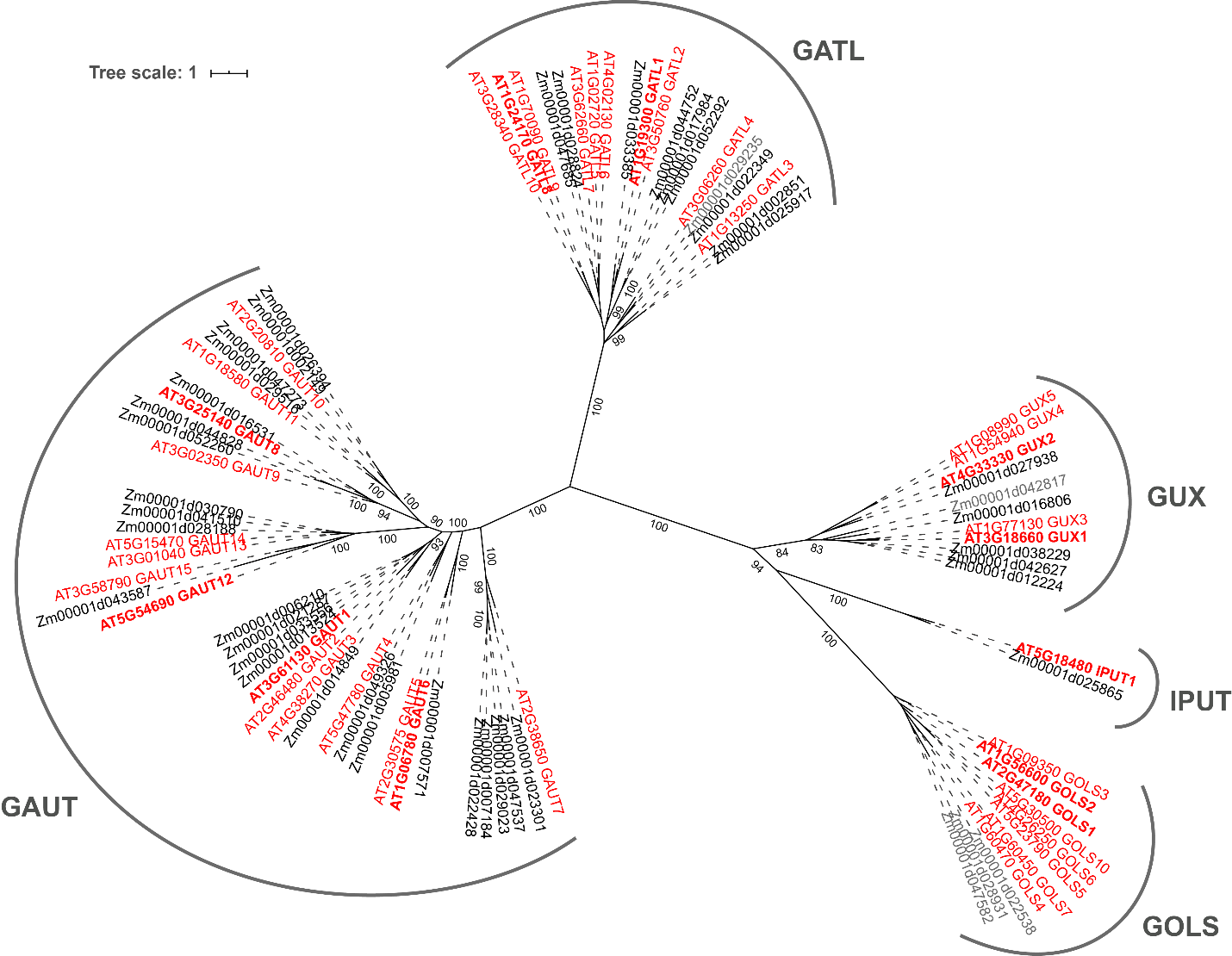


**Fig. S6** Dendrogram of plant GT8 genes. Names of genes labeled by black and red correspond to maize and *Arabidopsis* genes respectively. Grey color indicates maize genes that were not expressed in maize primary root. Names of genes encoding characterized proteins are in bold. Numbers indicate values of ultrafast bootstrap branch support for some branches


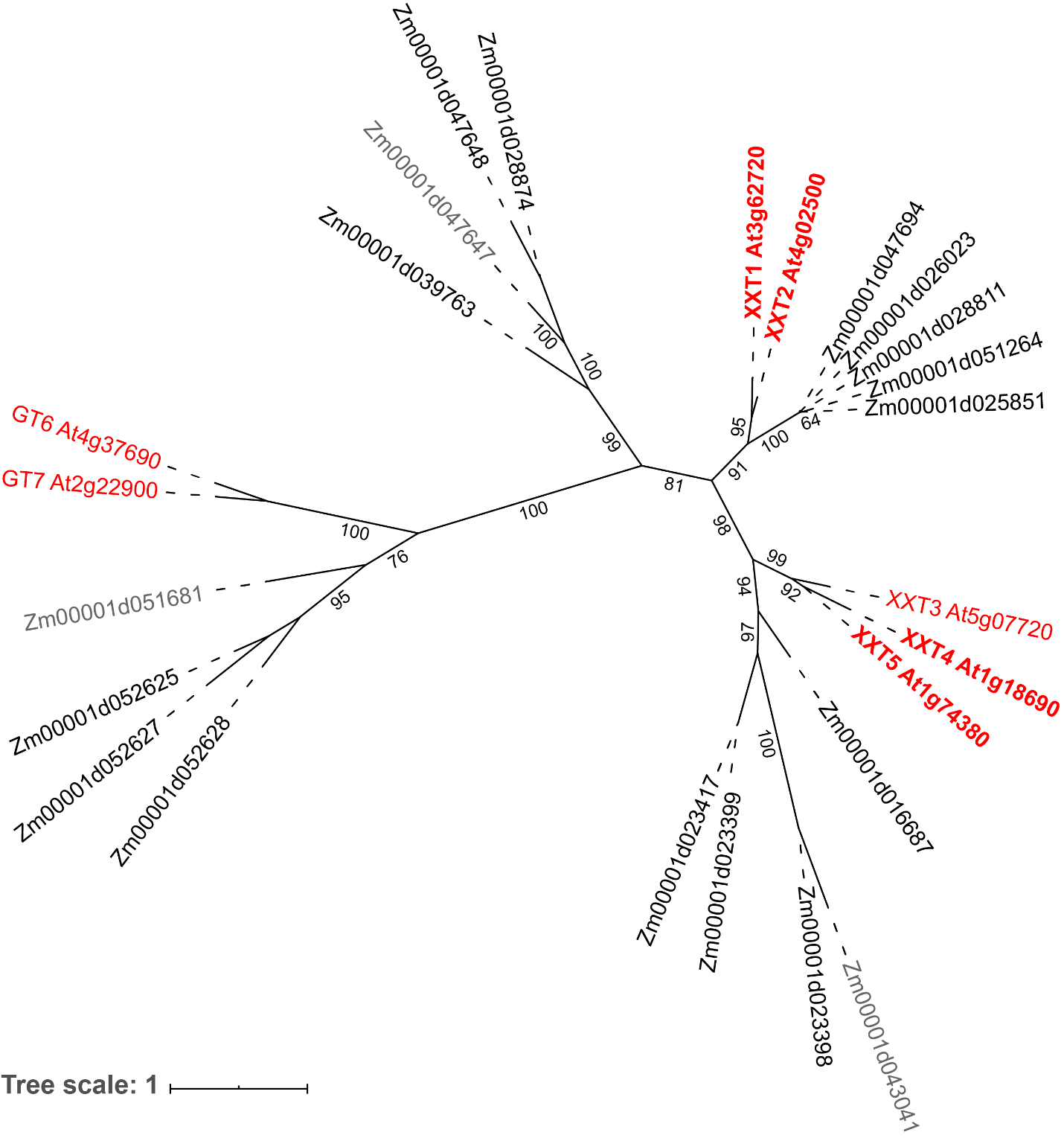


**Fig. S7** Dendrogram of plants GT34 genes. Black and red gene names correspond to maize and *Arabidopsis* genes respectively. Grey color indicates maize genes that were not expressed in maize primary root. Genes encoding characterized *Arabidopsis* XXT genes are in bold. Numbers are the ultrafast bootstrap branch support that shown for some branches


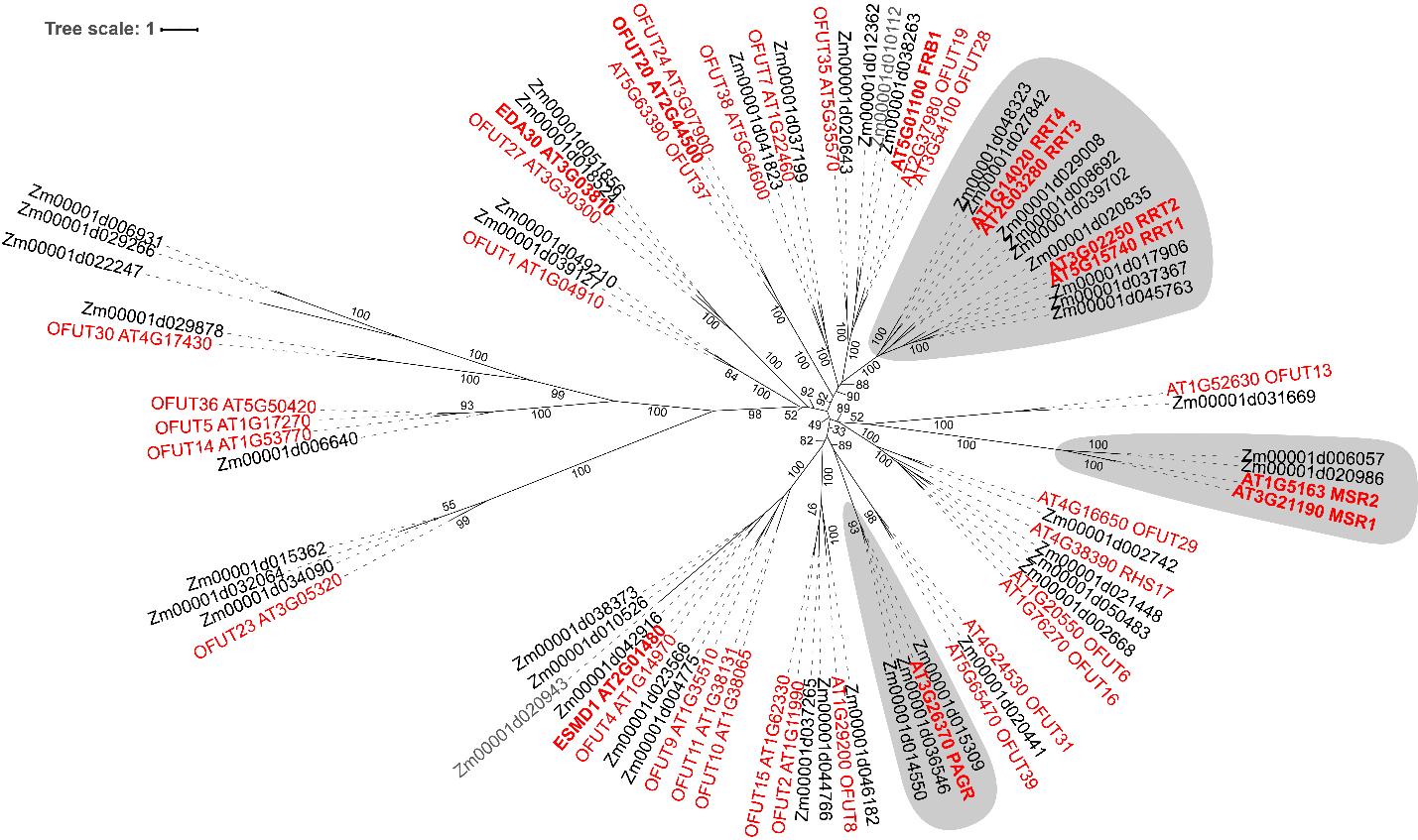


**Fig. S8** Dendrogram of plant GT106 genes. Black and red gene names correspond to maize and *Arabidopsis* genes respectively. Grey color indicates maize genes that were not expressed in maize primary root. Genes encoding characterized *Arabidopsis* genes are in bold. Numbers are the ultrafast bootstrap branch support that shown for some branches


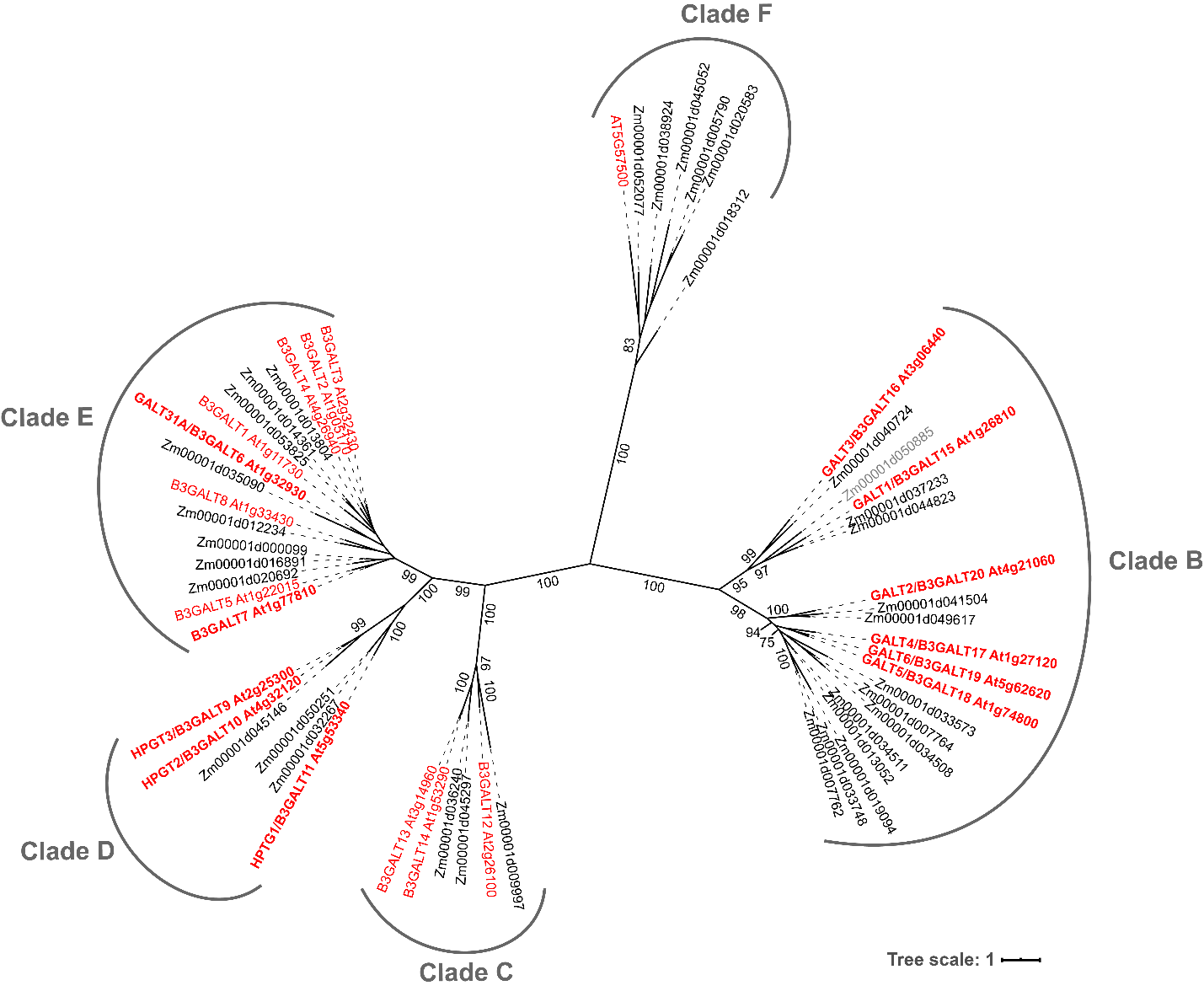


**Fig. S9** Dendrogram of plant GT31 genes. Black and red gene names correspond to maize and *Arabidopsis* genes respectively. Grey color indicates maize genes that were not expressed in maize primary root. Genes encoding characterized *Arabidopsis* GALT genes are in bold. Numbers are the ultrafast bootstrap branch support that shown for some branches


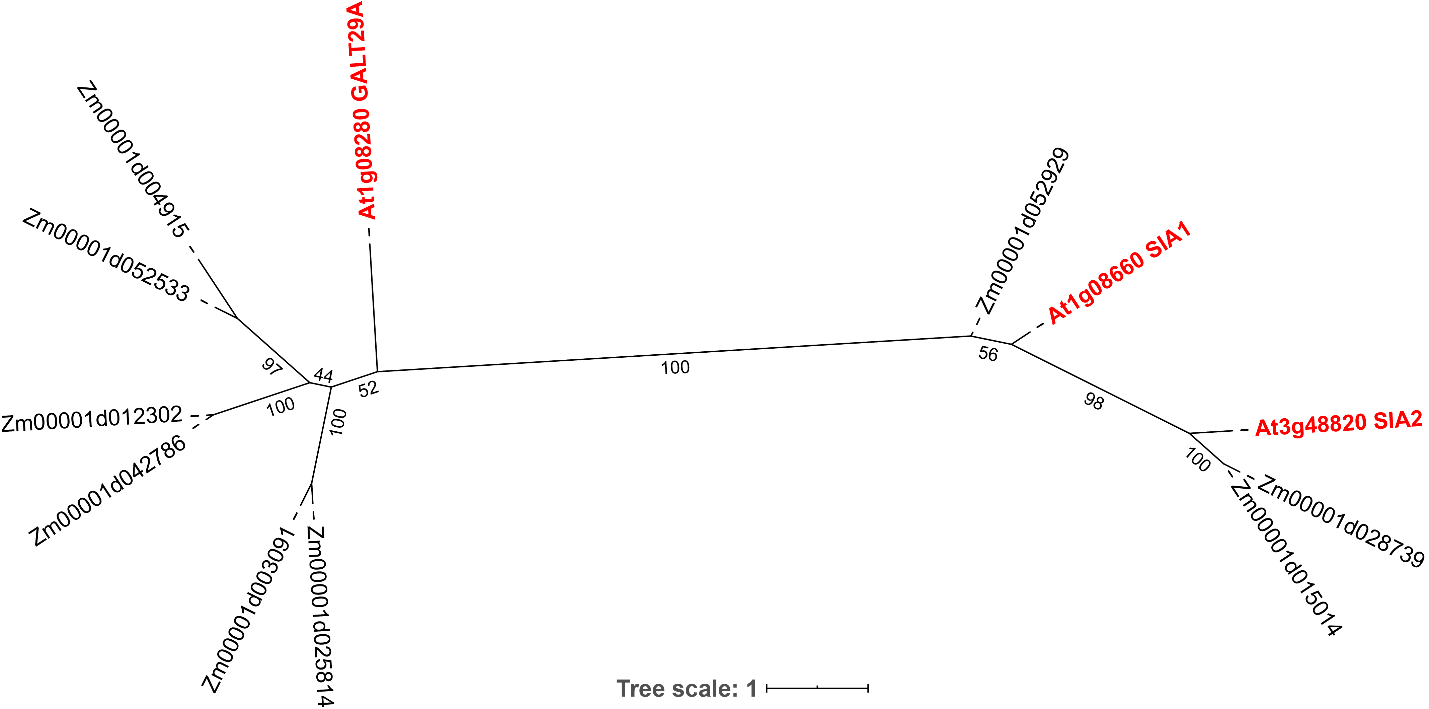


**Fig. S10** Dendrogram of plant GT29 genes. Black and red gene names correspond to maize and *Arabidopsis* genes respectively. Grey color indicates maize genes that were not expressed in maize primary root. Genes encoding characterized *Arabidopsis* GALT and SIA genes are in bold. Numbers are the ultrafast bootstrap branch support that shown for some branches

**References**

1 Penning, B. W., McCann, M. C. & Carpita, N. C. Evolution of the Cell Wall Gene Families of Grasses. *Frontiers in Plant Science* **10**, doi:ARTN 1205

10.3389/fpls.2019.01205 (2019).

2 Richmond, T. A. & Somerville, C. R. The cellulose synthase superfamily. *Plant Physiology* **124**, 495-498, doi:DOI 10.1104/pp.124.2.495 (2000).

3 Wang, L. Q. *et al.* Expression profiling and integrative analysis of the CESA/CSL superfamily in rice. *Bmc Plant Biol* **10**, doi:Artn 282

10.1186/1471-2229-10-282 (2010).

4 Chiniquy, D. *et al.* Three novel rice genes closely related to the Arabidopsis IRX9, IRX9L, and IRX14 genes and their roles in xylan biosynthesis. *Frontiers in Plant Science* **4**, doi:ARTN 83

10.3389/fpls.2013.00083 (2013).

5 Lee, C., Teng, Q., Zhong, R., Yuan, Y. & Ye, Z. H. Functional roles of rice glycosyltransferase family GT43 in xylan biosynthesis. *Plant Signal Behav* **9**, e27809, doi:10.4161/psb.27809 (2014).

6 Xu, H. *et al.* Genome-Wide Analysis of Sorghum GT47 Family Reveals Functional Divergences of MUR3-Like Genes. *Frontiers in Plant Science* **9**, doi:ARTN 1773

10.3389/fpls.2018.01773 (2018).

7 Liu, L. F., Paulitz, J. & Pauly, M. The Presence of Fucogalactoxyloglucan and Its Synthesis in Rice Indicates Conserved Functional Importance in Plants. *Plant Physiology* **168**, 549-+, doi:10.1104/pp.15.00441 (2015).

8 Anders, N. *et al.* Glycosyl transferases in family 61 mediate arabinofuranosyl transfer onto xylan in grasses. *P Natl Acad Sci USA* **109**, 989-993, doi:10.1073/pnas.1115858109 (2012).
