# Supplementary material for: Elongating maize root: zone-specific combinations of polysaccharides from type I and type II primary cell walls
Source: Sci Rep. 2020 Jul 2;10:10956. doi: 10.1038/s41598-020-67782-0 (PMC7331734; doi:10.1038/s41598-020-67782-0)
Supplement: Supplementary file 3 — Supplementary file3 [file 41598_2020_67782_MOESM3_ESM.docx]

**Elongating maize root: zone-specific combinations of polysaccharides from type I and type II primary cell walls /** Plant Molecular Biology / Liudmila V. Kozlova*, Alsu R. Nazipova, Oleg V. Gorshkov, Anna A. Petrova, and Tatyana A. Gorshkova / Kazan Institute of Biochemistry and Biophysics, FRC Kazan Scientific Center of RAS, Laboratory of Plant Cell Growth Mechanisms, Kazan, Russian Federation / * corresponding author: [kozlova@kibb.knc.ru](mailto:kozlova@kibb.knc.ru)

Table S2. Antibodies used in the current study

| **Antibody** | **Immunogene** | **Target polysaccharide** | **Manufacturer, country** | **Reference** |
| --- | --- | --- | --- | --- |
| BG1 | Mixed-linkage glucan oligosaccharides | Mixed-linkage glucan | Biosupplies Australia Pty Ltd, Australia | Meikle et al., 1994 |
| AX1 | Arabinoxylan oligosaccharides | Arabinoxylan | INRA, France | Guillon et al., 2004 |
| LM27 | Complex pectic immunogen | Grass heteroxylan or associated molecule | University of Leeds, United Kingdom | Cornuault et al. (2015) Planta 242, 1321-1334 |
| LM28 | Complex pectic immunogen | Glucoronoxylan | University of Leeds, United Kingdom | Cornuault et al. (2015) Planta 242, 1321-1334 |
| LM11 | Xylooligosaccharides | Xylan | University of Leeds, United Kingdom | McCartney et al., 2005 |
| LM25 | Galactosylated oligosaccharides of xyloglucan | Xyloglucan | University of Leeds, United Kingdom | Pedersen et al. (2012) J. Biol. Chem. 287, 39429-39438 |
| LM20 | Arabidopsis seed mucilage | Methyl-esterified homogalacturonan | University of Leeds, United Kingdom | Verhertbruggen et al. (2009) Carbohydr. Res. 344, 1858-1862. |
| LM19 | Apple fruit pectic galacturonan | Un-esterified homogalacturonan | University of Leeds, United Kingdom | Verhertbruggen et al. (2009) Carbohydr. Res. 344, 1858-1862. |
| RU2 | RG-I oligosaccharides | RG-I | INRA, France | Ralet et al., Planta (2010) 231:1373–1383 |
| LM5 | Galactotetraose | (1,4)-β-d-galactans | University of Leeds, United Kingdom | Jones et al. (1997) Plant Physiol. 113, 1405-1412. |
| LM26 | Complex pectic immunogen | (1,6)-galactosyl substitution in (1,4)- β-d-galactans | University of Leeds, United Kingdom | Torode et al. (2018) Plant Physiol. 176, 1547-1558 |
| LM6 | Arabinoheptaose | (1,5)-α-l-arabinans | University of Leeds, United Kingdom | Verhertbruggen et al. (2009) Plant J. 59, 413-425. |
